# Spike-Stalk Injection Method Causes Extensive Phenotypic and Genotypic Variations for Rice Germplasm

**DOI:** 10.3389/fpls.2020.575373

**Published:** 2020-09-25

**Authors:** Yuanyi Hu, Bigang Mao, Yumei Xia, Yan Peng, Dan Zhang, Li Tang, Ye Shao, Yaokui Li, Bingran Zhao

**Affiliations:** ^1^ State Key Laboratory of Hybrid Rice, Hunan Hybrid Rice Research Center, Changsha, China; ^2^ Molecular Breeding Laboratory, National Innovation Center of Saline-Alkali Tolerant Rice in Sanya, Sanya, China; ^3^ Long Ping Branch, Graduate School of Hunan University, Changsha, China; ^4^ College of Agricultural, Hunan Agricultural University, Changsha, China

**Keywords:** rice, genome sequencing, genetic diversity, exogenous DNA transfer, quantitative trait locus mapping

## Abstract

Genetic diversities or favorable genes within distantly related species are the important resources for crop genetic improvement and germplasm innovation. Spike-Stalk injection method (SSI) has long been applied in rice genetic improvement by directly introducing genetic materials from non-mating donor species, while its inheritance patterns and the underlying mechanisms are poorly elucidated. In this study, a rice variant *ERV1* with improved yield-related traits was screened out in the way of introducing genomic DNA of *Oryza eichingeri* (2n=24, CC genome) into *RH78* (*Oryza sativa L*. 2n=24, AA genome) using SSI method. Genome-wide comparison revealed that the genomic heterozygosity of *ERV1* was approximately 8-fold higher than *RH78*. Restriction-site associated DNA sequencing technology (RAD-seq) and association analysis of the *ERV1* inbred F2 population identified 5 quantitative trait loci (QTLs) regions responsible for these yield-related traits, and found that genomic heterozygosity of *ERV1* inbred lines was significantly lower than *ERV1*, while spontaneous mutation rate of the *ERV1* inbred lines was significantly higher than *ERV1*. Our results preliminarily uncovered the inheritance patterns of SSI variant rice, and the potential genomic regions for traits changes, which yielded novel insights into the mechanisms of SSI method, and may accelerate our understanding of plant genome evolution, domestication, and speciation in nature.

## Introduction

Rice (*Oryza sativa L*.) is one of the most important food crops in the world, providing 21% of the total calorie intake of the global population and up to 76% of calorie intake in Southeast Asia(Fitzgerald et al., 2009; Chen, 2010). Increasing rice yield is therefore of great importance to global food security. Two breakthroughs in rice yield that led to major increases in rice productivity over the last century (Yuan and Virnani, 1989) include the application of semi-dwarf rice varieties in the 1950s–1960s (Peng, 1994; Ashikari et al., 2005), and the development of Three-line and Two-line hybrid rice (Zhou et al., 2012; Chen and Liu, 2014), which were commercially developed in the 1970s and 1990s, respectively. These breakthroughs were mainly due to the discovery and application of new genes or novel alleles from wild rice species (Brar and Khush, 1997; Zhao et al., 2007). Genetic diversities or favorable genes from distantly related species are therefore crucial for genetic improvement and germplasm innovation in rice, and can help guarantee the sustained growth of rice yield in the future (Gill et al., 2011).

Transferring genetic materials from wild relatives of rice into modern cultivated rice is a promising way to use genetic information and favorable genes (Zamir, 2001; Wang et al., 2017). In the genus *Oryza*, the wild species can be used to genetically improve rice because they have abundant genetic variation with many agronomical useful genes (Chen, 2010; Jena, 2010). Generally, researchers have relied on distant hybridization to introduce “exotic” allelic diversity, or on genetic engineering to introduce “foreign” genes (Zhou et al., 1983; Zhao et al., 2005) from wild rice. Over the last few decades, several genes, or QTLs controlling traits of economic importance, such as cytoplasmic male sterility (CMS), yield-related QTLs, resistance of bacterial leaf blight, blast and brown plant-hopper, have been tagged or cloned (Wang et al., 2005), and transferred from wild rice into elite rice cultivars by means of distant hybridization and genetic engineering. However, several incompatibility barriers such as low crossability, linkage drag, and limited recombination between chromosomes of wild and cultivated species limit the application of distant hybridization. The limited number of cloned genes or QTLs controlling traits of economic importance, and the security of genetically modified food also hampered the development and large-scale application of genetic engineering.

In the past three decades, a large number of new crop germplasms with improved characteristics in yield, plant type, grain quality, and stress tolerance have been created in China (Hu et al., 2014; Peng et al., 2016), benefiting from a widely used series of methods called Exogenous DNA Transfer (EDT) methods, mainly including pollen tube pathway method (Zhou et al., 1983; Wang et al., 2005), seed immersion method (Luo and Wu, 1989), and the Spike-Stalk Injection method (de la Peña et al., 1987; Zhao et al., 2007). Researchers previously proved that genomic DNA of wild species could be introduced into the rice panicle at the differentiation stage, the ovary at the meiosis stage, or into dry seeds at the water absorbency stage (Zhao et al., 2007). Zhao et al. (2005) analyzed a genetically stable variant, *YVB*, that was developed from a mutant of *V20B* (a rice maintainer line) through transformation of genomic DNA of wild rice (*Oryza minuta*). They found high DNA polymorphisms between *YVB* and *V20B*, and preliminary confirmed the integration of special DNA fragment from *O. minuta* in *YVB* genome. Meanwhile, they identified the genes responsible for the trait changes in *YVB* relative to *V20B*, and revealed that allelic variation might be the main factor contributing to the abundant phenotypic changes of this variant (Peng et al., 2016). However, the possible molecular mechanisms underlying trans-species transfer of genetic information, especially genome-wide genetic variation and inheritance of variants in the early generations compared to the recipients, are poorly elucidated.

Comparative genomic analyses among closely related species have allowed for insight into plant gene and genome evolution (Bennetzen, 2007; Paterson et al., 2010). For instance, studies of yeast, *Drosophila*, and human genomes have revealed mechanisms of gene and genome evolution in fungi and animals (Scannell et al., 2006; Clark et al., 2007). Meanwhile, comparison of *O. nivara*, *O. glaberrima*, *O. barthii*, *O. glumaepatula*, and *O. meridionalis* genomes unveiled how morphological and reproductive diversity have arisen in specific lineages as a result of expansions or contractions of gene families, providing more evidence for the processes of adaptation and speciation (Zhang et al., 2014). However, current comparative genomic analyses mainly focus on the comparison between different varieties, or between closely related species of flowering plants; few are carried out to query the fundamental rules of inheritance from the parent to the offspring by means of horizontal and/or vertical transmission, especially in the early generations.

In this study, we conducted whole-genome sequencing and comparative genomic analysis of *ERV1*, an original variant with greatly improved yield traits, and an inbred *ERV1* population. *ERV1* was derived from an *indica* variety *RH78* by transferring genomic DNA of *O. eichingeri* to the recipient rice *RH78* through the SSI method. Our results would accelerate the process for unveiling the possible mechanisms underlying SSI method, and may enhance our understanding of plant genome evolution, domestication, and speciation in nature.

## Materials and Methods

### Spike-Stalk Injection Method

SSI method is a modified technique based on the procedure invented by *Pena* (de la Peña et al., 1987). To perform the technique, we waited until recipient rice had just undergone meiosis (up to 2–4 days after meiosis) and selected spikes that had a distance of 1–5 cm from the petiole of the flag leaf to the top of the 2nd leaf. Part of the leaf sheath of the top 3rd leaf was removed, and the uppermost internode was exposed. Donor DNA (50–100 μl) at a concentration of about 500 ng/μl was mixed with 0.2×SSC solution, and 0.02 M CaCl_2_ solution was then injected into the internode with a microsyringe at an inclined angle of about 30–45°. The injected spike was immediately bagged with parchment (to avoid the pollution of exogenous pollen) until harvest. The harvested seeds were planted uniparted. From initial heading stage to the maturity stage, rice plants with significant trait differences from recipient rice were considered variant rice.

### Plant Materials and Traits Measurement

The variant *ERV1* was discovered in the experimental field of the Hunan Hybrid Rice Research Center, Changsha, in September 2013. The recipient rice *RH78* was planted in the same field at the same time, and with the same management to compare agronomic traits. *ERV1* was selfed to generate the F2 selfing population. *ERV1*, five mature plants of *RH78*, and 216 *ERV1* inbred F2 lines were sampled to determine mean values of yield characters, including heading stage (HS), flag leaf length (FLL), flag leaf width (FLW), plant height (PH), effective tiller number (TN), main panicle length (PL), spikelet per panicle (SP), seed setting rate (SSR), and 100-grain weight (GW). HS refers to the time from seed sowing to first-earing phase. FLL and FLW represent the maximum length and width of the flag leaf measured by an electronic digital caliper. PH refers to the distance from the ground to the highest point of one plant. TN is the number of tillers that can develop mature panicles. SP is the spikelet number of the main panicle. SSR is the number of filled grains divided by the spikelet number of the main panicle. GW refers to the weight of 100 filled grains of the main panicle. Student’s *t*-tests were used for all trait data of *ERV1* and *RH78* to identify significant differences.

### DNA Extraction and Genome Sequencing

Genomic DNA of *ERV1*, *RH78*, and 216 *ERV1* inbred F2 lines were extracted following the standard procedure of a PureLink™ Genomic Plant DNA Purification Kit (Invitrogen). For genome re-sequencing, the genomic DNA of *RH78* and *ERV1* were extracted, and then fragmented randomly. The DNA fragments of desired length (500 base pairs) were visualized with agarose gel electrophoresis, adapter primers were added to generate DNA clusters, and DNA clusters were subjected to HiSeq 2000 paired-end sequencing. For RAD-seq of *ERV1* inbred F2 lines, the genomic DNA of each of the 216 inbred lines was digested with the TaqI restriction endonuclease, and DNA fragments between the sizes of 300 and 700 bp were isolated with agarose gel electrophoresis. Adapters were ligated to the isolated DNA fragments for cluster preparation, and then subjected to the HiSeq 2000 platform for 100 base paired-end sequencing (Davey et al., 2011).

### Bioinformatics Analysis Process for Re-Sequencing

The raw data generated by the HiSeq 2000 sequencer was subjected to a series of filters to generate a high-quality data set (Huang et al., 2009; Gu et al., 2013). The high-quality data was then aligned to the *Nipponbare* genome (allowing three mismatches) *in silico* using the SOAPaligner software (Yu et al., 2001; Ouyang et al., 2005). Sequencing coverage and depth of each sample were calculated based on the alignment result. Based on the consensus sequence between the reference and each of the sequenced samples, single nucleotide polymorphisms (SNPs) in *ERV1*, *RH78*, and each of the *ERV1* F2 inbred lines relative to the *Nipponbare* genome were identified using the SOAPsnp software with the following filtering steps: mapping quality > 20; coverage depth between 1 and 300; distance of adjacent variation > 5 bp; and copy number < 2. According to the filtered SNPs set, SNPs between *ERV1* and *RH78* were further identified with the following filter steps: target mapping quality > 20; the sequencing depth of homologous site should be > 2; and the sequencing depth of heterozygous sites should be > 6. The final SNP set was then annotated according to the reference genome sequence of the *japonica* cultivar *Nipponbare*.

### Bioinformatics Analysis Process for RAD-Seq

The sequencing reads from each line were obtained according to the sequence tag, and then mapped to the *Nipponbare* genome sequence for alignment using the SOAPaligner software (Gu et al., 2013). The results of SOAP alignment were then transformed and subjected to SAMtools (Li et al., 2009) to generate the input files suitable for realSFS software (version 0.983). Nucleotide sites with the likelihood of a genotype for each line with a probability higher than 0.95 and a total population depth higher than 40 were extracted as candidate SNPs. The recombination breakpoints of the F2 population were then identified according to the method developed by the research group of Huang et al. (2009) with some modification (Duan et al., 2013). The Bin map of each line was constructed using a PERL script, and QTLs were identified by composite interval mapping using MapQTL5 software.

### Estimate of Genomic Heterozygosity and Spontaneous Mutation Rate

Mutation sites in *ERV1* refer to the genotype in *ERV1* that was different from the genotype of *RH78*. For example, if the genotype of *RH78* was A, and *ERV1* was homologous G, or C, or T, this genotype was defined as a homologous SNP; if the *ERV1* was heterozygous A/G, A/C, A/T, this genotype was defined as a heterozygous SNP; if the genotype of *ERV1* was T/C, or T/G, or C/G, we defined it as a mutation SNP. The genomic heterozygosity was calculated as the number of heterozygous SNPs divided by the length of covered bases for the sequencing sample. One site where the genotype of >20 F2 line was not identical to the genotype of *RH78* and/or *ERV1* was defined as a spontaneous mutation. The spontaneous mutation rate of the *ERV1* inbred line was calculated as the number of spontaneous mutations (>20 lines) in the F2 population divided by the length of covered bases for the sequencing sample.

## Results

### Trait Comparison Between *ERV1* and *RH78*

**Figure 1:**
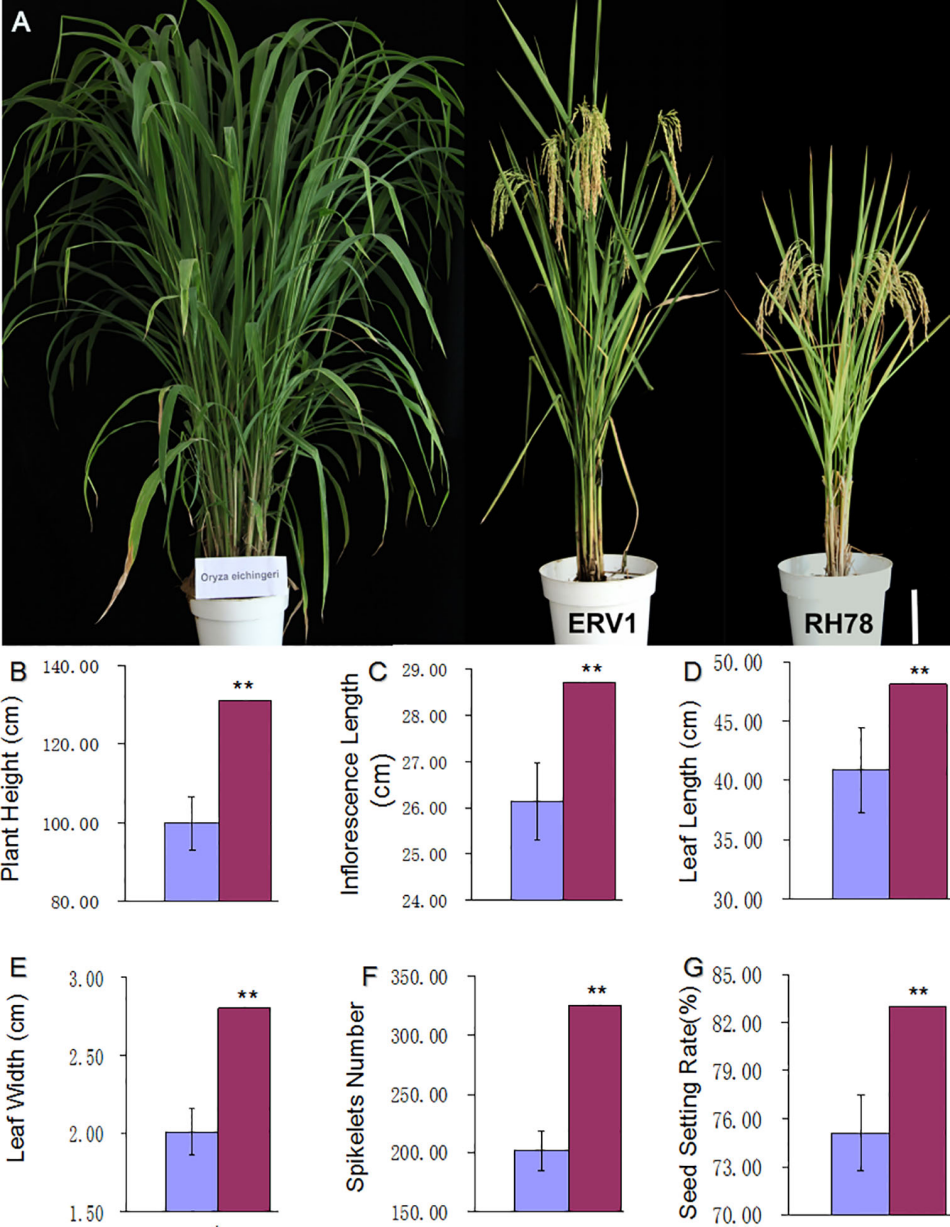
Trait comparison of *ERV1* and *RH78*. **(A)** Plant phenotype of the donor rice *O. eichingeri* (left), the recipient rice *RH78* (right), and the variant rice *ERV1* (middle), the white bar = 20 cm. **(B–G)** is the comparison of main agronomic characters of *RH78* and *ERV1* in the mature stage; **(B)** plant height, **(C)** inflorescence length, **(D)** flag leaf length, **(E)** flag leave width, **(F)** spikelet number, and **(G)** seed setting rate. The main agronomic characters of RH78 and ERV1 were compared and analyzed by one-way ANOVA method, and the symbol “**” indicate significant difference detected by LSD tests at P < 0.01.

A large proportion of traits in *ERV1* were the intermediate forms between the recipient *RH78*, and the donor *O. eichingeri*. This made ERV1 seems to be a hybrid F1 cross between *RH78* and *O. eichingeri* (**Figure 1A**). We surveyed the main agronomic characters of *RH78* and *ERV1* in the mature stage and found that *ERV1* had higher PH (**Figure 1B**), longer inflorescence length (**Figure 1C**), longer and wider flag leaves (**Figures 1D, E**), larger panicles (**Figure 1F**), and a higher SSR (**Figure 1G**) compared to *RH78*. The spikelet number and SSR of *ERV1* were significantly higher than *RH78*, while the TN and GW of *ERV1* were almost equal to *RH78* (**Table S1**), meaning that *ERV1* has a higher yield potential in breeding practice.

### Genome Sequencing of *ERV1* and *RH78*

Using Illumina sequencing technology with a whole-genome shotgun sequencing approach, a total of 11.4 and 10.2 G bases (Gb) of raw sequencing data were generated for *ERV1* and *RH78*, respectively (Raw sequence data of *ERV1* and *RH78* have been deposited in the NCBI Short Read Archive with access number SRA7923627). After a series of corrections and filters, 10.81 Gb and 10.10 Gb clean data for *ERV1* and *RH78*, respectively, were retained for downstream analysis. The clean data were then aligned to the *Nipponbare* genome, a high-quality rice reference genome with an effective genome size of 372,317,567 base pairs (bp), for whole-genome comparison using the SOAPaligner software algorithm (SOAP2, version 2.20). The *ERV1* and *RH78* clean data covered 312,397,330 bp (83.66%) and 303,082,602 bp (81.06%) of the *Nipponbare* reference genome, providing an effective sequencing depth of 29.03-fold for *ERV1* and 27.14-fold for *RH78* (**Table 1**).

**Table 1:** Statistics of the sequencing data of *ERV1* and *RH78*.

Sample	Reference length (bp)	Raw data (Gb)	Clean data (Gb)	Covered base (bp)	Sequencing depth (fold)	Coverage (%)
*RH78*	372,317,567	10.31	10.10	303,082,602	27.14	81.06
*ERV1*	372,317,567	11.41	10.81	312,397,330	29.03	83.66

Reference length refers to the assembled chromosome length of the Nipponbare genome (IRGSPv6).

### Identification and Annotation of SNPs

The wide genomic coverage and high sequencing depth of *ERV1* and *RH78* sequencing data were considered suitable for use in the identification of single nucleotide polymorphisms (SNPs). After a series of corrections and filtering steps, a total of 1,397,635 SNPs, including 1,257,597 homozygous SNPs (homo-SNPs), and 101,822 heterozygous SNPs (hetero-SNPs) were detected in *RH78* compared to *Nipponbare* genome sequence. Hetero-SNPs accounted for 7.29% of the total SNPs, and the genomic heterozygosity of *RH78* genome was 3.36 × 10^-4^ (**Table S2**). For *ERV1*, 1,898,506 SNPs, including 1,087,532 homo-SNPs and 810,974 hetero-SNPs were identified relative to *Nipponbare* genome. The proportion of hetero-SNPs was 42.72% of the total SNPs, and the genomic heterozygosity of *ERV1* was as high as 25.96 × 10^-4^ (**Table S3**), approximately 8-fold higher than that of *RH78*.

**Figure 2:**
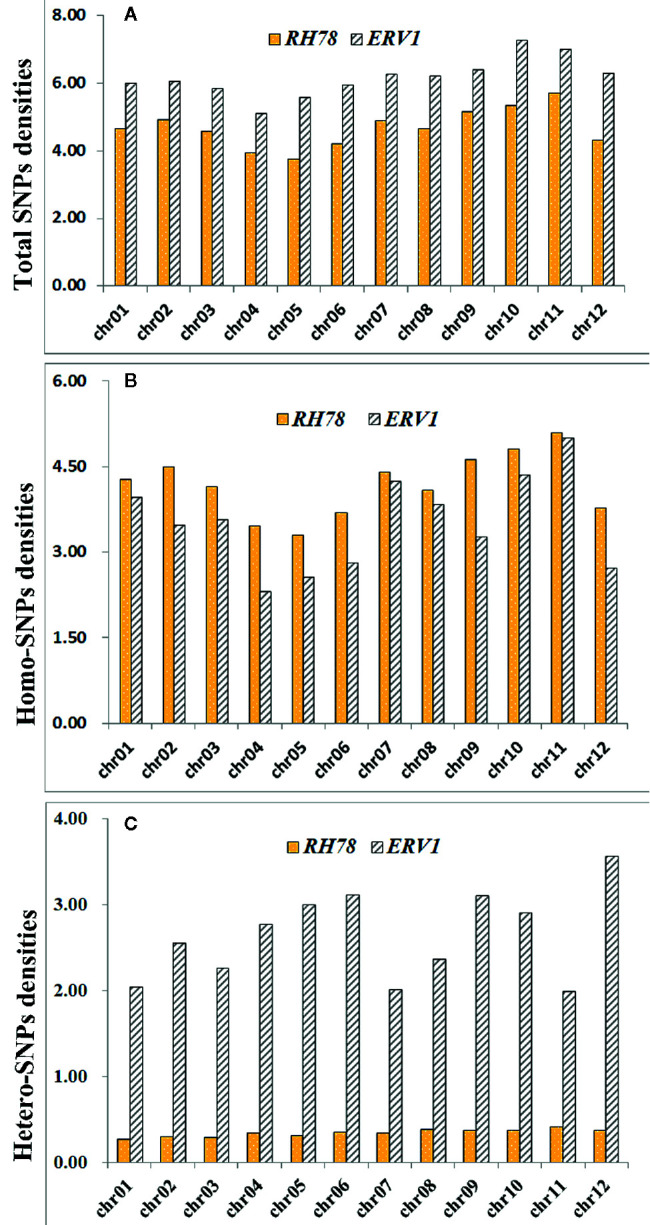
Distribution of single nucleotide polymorphism (SNP) densities of *ERV1* and *RH78* compared to the *Nipponbare* genome in all chromosomes. **(A)** Total SNPs densities of *RH78* (yellow bars) and *ERV1* (gray bars) in 12 chromosomes; **(B)** homo-SNPs densities of *RH78* (yellow bars) and *ERV1* (gray bars) in 12 chromosomes; **(C)** hetero-SNPs densities of *RH78* (yellow bars) and *ERV1* (gray bars) in 12 chromosomes. All horizontal axes of **(A–C)** represent the chromosome number, and the vertical axes of **(A–C)** represent the SNP densities on each chromosome.

**Figure 3:**
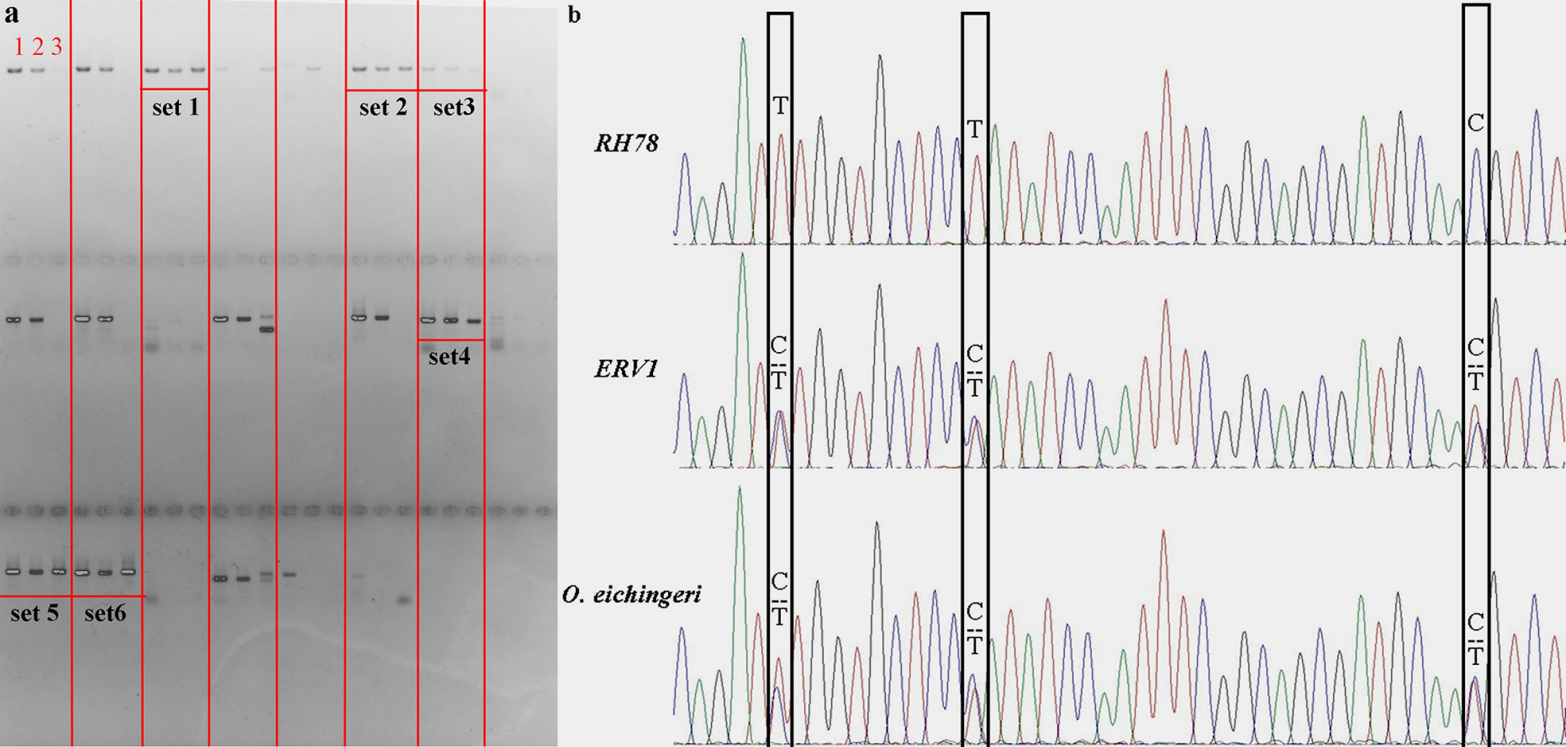
PCR amplification, Sanger sequencing and comparison of the homologous sequences among *RH78*, *ERV1*, and *O. eichingeri*. **(A)** PCR amplification of 24 randomly selected DNA fragments in *RH78*, *ERV1*, and *O. eichingeri*. The PCR bands in each set were presented with the same order as number 1 for RH78, number 2 for ERV1 and number 3 for *O. eichingeri*. The 6 DNA fragments that were simultaneous amplified in *RH78*, *ERV1* and *O. eichingeri* were noted as set1, set2, set3, set4, set5, and set6, and only PCR products of set4 were sequenced with good quality. **(B)** Result of Sanger sequencing and comparison of the set4 sequences. The single nucleotide polymorphisms (SNPs) or mutations among *RH78*, *ERV1*, and *O. eichingeri* were labeled with black boxes.

The distribution patterns of SNPs in *ERV1* and *RH78* genomes were presented using the SNP density, which was calculated as the total number of SNPs divided by the total covered bases in each chromosome. The average total SNP density across the whole genome was 4.65 SNPs/kb in the *RH78* genome (**Table S2**), slightly lower than 6.15 SNPs/kb in the *ERV1* genome (**Table S3**). While, the density of hetero-SNPs in *ERV1* (2.64 hetero-SNPs/kb) was much higher than that in *RH78* (0.34 hetero-SNPs/kb). At the chromosome level, the highest density of total SNPs and homo-SNPs was identified in chromosome 11 for both *RH78* and *ERV1*, and the lowest in chromosome 5 for *RH78* and chromosome 4 for *ERV1*. The highest density of hetero-SNPs was discovered in chromosome 11 for *RH78*, but in chromosome 12 for *ERV1*, and the lowest in chromosome 1 for *RH78* and chromosome 11 for *ERV1* (**Figure 2**). To answer whether these SNPs in *ERV1* came from O. *eichingeri*, 2 DNA fragments (less than 1000 bp) in each chromosome of *Nipponbare* genome that were simultaneous covered by the sequencing reads of *RH78*, ERV1, and *O. eichingeri* were randomly selected, but only 6 DNA fragments were simultaneous amplified in *RH78*, *ERV1*, and *O. eichingeri* using PCR amplification method (**Figure 3A**), of which only one DNA fragment was sequenced with good quality. Comparing the sequencing data of this DNA fragment among *RH78*, *ERV1*, and *O. eichingeri* found that 3 heterozygous SNPs in *ERV1* relative to *RH78* were totally identical to *O. eichingeri* (**Figure 3B**), which partially proved that these SNPs in *ERV1* came from *O. eichingeri*. However, more experiments are needed.

**Figure 4:**
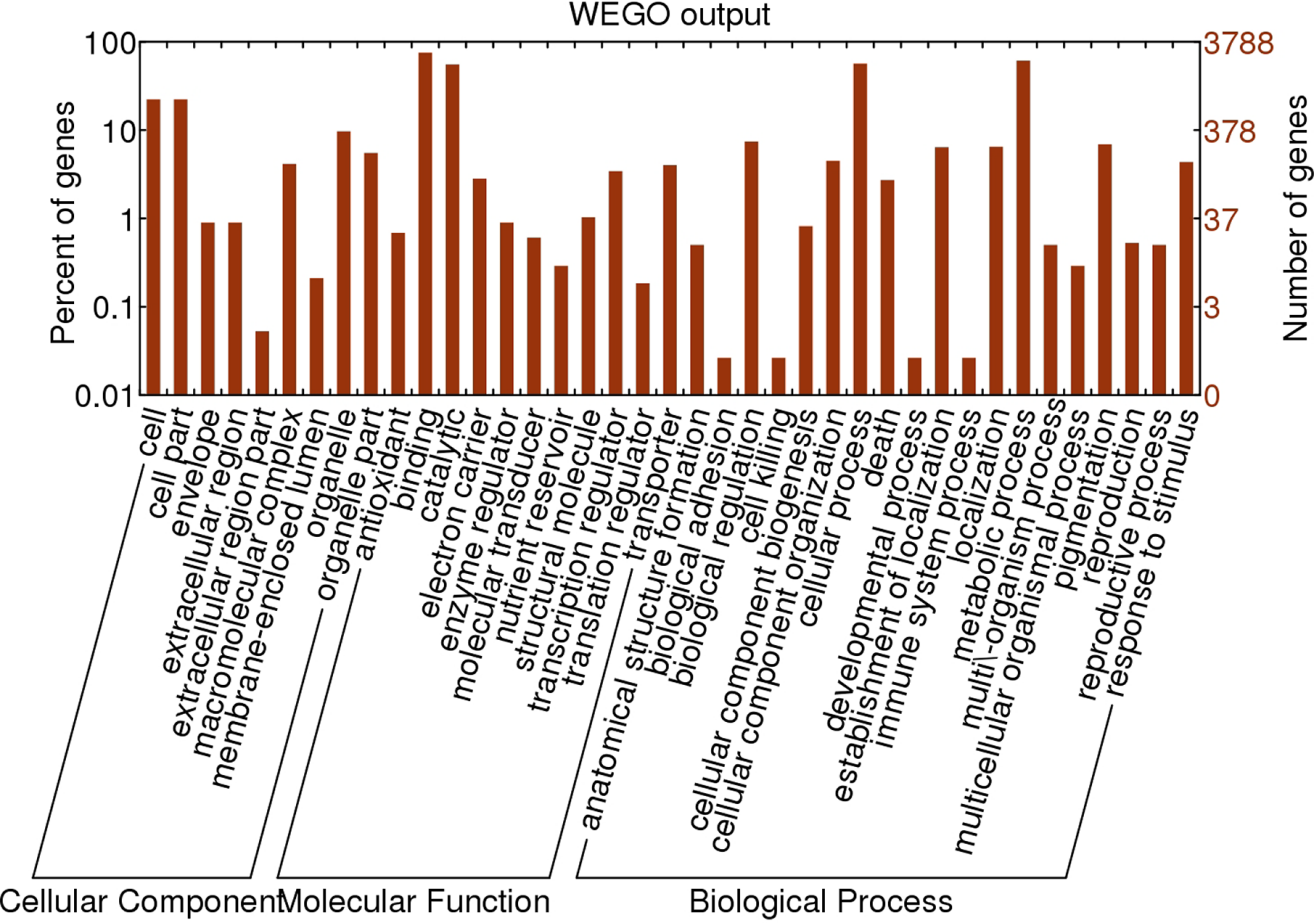
Gene Ontology (GO) classification of the genes affected by Non-synonymous hetero-SNPs in *ERV1* compared to *RH78*. The horizontal axis represents the GO items, including cellular component, molecular function, and biological process. The left vertical axis represents the percent of genes affected by Non-synonymous hetero-SNPs of the corresponding GO item, and the right vertical axis represents the number of genes affected by Non-synonymous hetero-SNPs of the corresponding GO item.

The genome-wide SNPs in *ERV1* compared to *RH78* were filtered out from the SNP data mentioned above. A total of 455,244 SNPs were identified in *ERV1*, of which 98.79% (449,726) SNPs were heterozygous (**Figure S1**); only 5,518 (1.21%) SNPs were homozygous (**Table 2**). The hetero-SNPs in *ERV1* were annotated according to the reference genome sequence of *japonica* cultivar *Nipponbare* because they were a very high proportion (98.79%) out of the total SNPs. After filtering, a total of 14,955 Non-synonymous hetero-SNPs (Ns-hetero-SNPs) that might affect the expression and function of the allele were identified in 7,325 genes (**Table S4**), of which 94.67% (6,934) genes had less than 5 Ns-hetero-SNPs. These genes were then functionally annotated using gene ontology (GO) classification (http://www.geneontology.org) and were categorized into 39 functional groups based on sequence homology. In each of the three main categories (biological process, molecular function, and cellular component) of the GO classification, there were 19, 11, and 9 functional groups, respectively (**Figure 4**).

**Table 2:** Statistics of single nucleotide polymorphisms (SNPs) in *ERV1* relative to *RH78*.

Chromosome	SNP number	Homo-SNPs	Hete-SNPs
chr01	43,225	343	42,882
chr02	50,240	738	49,502
chr03	45,020	495	44,525
chr04	42,737	656	42,081
chr05	48,879	391	48,488
chr06	51,818	564	51,254
chr07	20,626	236	20,390
chr08	24,943	303	24,640
chr09	34,147	518	33,629
chr10	29,288	464	28,824
chr11	16,024	225	15,799
chr12	48,297	585	47,712
Total	455,244	5,518	449,726

### Sequencing and Association Analysis of the *ERV1* Inbred F2 Population

Restriction-site associated DNA sequencing technology (RAD-seq) was carried out on the *ERV1* inbred F2 population. The RAD-seq data were then used to construct a genetic linkage map with high-density SNPs for use in association analysis exploring genotype-phenotype relationships at the whole-genome level.

RAD-seq libraries of 216 *ERV1* F2 inbred lines were constructed, yielding approximately 30.85 Gb raw sequencing data (**Table S5**, Raw sequence data of *ERV1* F2 inbred lines have been deposited in the NCBI Short Read Archive with access number SRA7923627), and providing an average sequencing coverage of 5.97%, and a mean sequencing depth of 3.23-fold for each F2 line. After manual filtration, a total of 32,644 population SNPs were selected for the F2 inbred population (**Table 3**). The distribution of the population SNPs along the chromosomes was uneven, with high-density and low-density regions (**Figure S2**). The genotype and bin information of each line of the F2 population was identified according to the recombination breakpoints. Using Joinmap 4.1 software, a total of 1,465 bins were used to construct the genetic map (**Table 3**). The size of the genetic map was 1489.7 centimorgans (**Figure S3**).

**Table 3:** Single nucleotide polymorphisms (SNPs) used for genotyping and bin number and genetic distance on 12 rice chromosomes for the F2 population.

Chromosome	Heterozygous SNPs in *ERV1*	Number of SNPs (population)		Number of bins	Genetic distance (cM)
		Before filtration	After filtration		
Chr01	42,881	7,523	2,816	169	234.8
Chr02	49,500	9,189	3,440	174	150.9
chr03	44,525	8,205	2,568	167	187.2
chr04	42,078	8,113	2,204	123	95.8
chr05	48,486	8,774	6,184	140	91.7
chr06	51,254	9,763	3,151	149	98.7
chr07	20,390	4,424	977	75	126.5
chr08	24,640	4,953	1,078	81	126.8
chr09	33,629	6,721	2,724	93	92.2
chr10	28,824	6,091	2,329	86	78.2
chr11	15,799	3,539	1,207	83	104.5
chr12	47,711	8,991	3,966	125	102.4
Total	449,717	86,286	32,644	1,465	1,489.7

According to the genetic map and the phenotypic data of the F2 population (**Table S6**), gene mapping of the 9 yield-related traits was carried out in QTLcartographer software using the composite interval mapping method. A total of 5 QTLs were detected (**Table 4**). The numbers of QTLs, as well as their contributions to phenotypic variance are summarized in **Table 4**. One QTL region, located at bin22, had an LOD value of 4.1, explained 8.4% of the PH variation, and was detected on chromosome 1. One QTL for PL was discovered on chromosome 2; it had a LOD score of 3.55 and explained 7.3% of the phenotypic variation. One QTL for GW was identified on chromosome 4, located at bin 525, had a LOD score of 3.55, and explained 7.3% of the phenotypic variation. Two QTLs for TN were discovered on chromosome 2, and 11, separately. The first QTL was located at bin260, had an LOD score of 3.9, and explained 8% of the phenotypic variation; the other QTL for TN was located at bin1207, had an LOD score of 3.53, and explained 6.8% of the phenotypic variation.

**Table 4:** Summary of quantitative trait loci (QTL) information of all traits.

Trait	Chromosome	Peak bin	Start	End	Bin length (bp)	LOD	R2 (%)
PH	chr01	bin22	7779967	8059948	279,981	4.1	8.4
PL	chr02	bin263	25718520	25761663	43,143	3.55	7.3
GW	chr04	bin525	19108032	19172986	64,954	3.53	7.3
TN	chr02	bin260	25424655	25516358	91,703	3.9	8
	chr11	bin1207	15862580	16106680	244,100	3.53	6.8

### Genomic Heterozygosity and Spontaneous M in the *ERV1* Inbred F2 Lines

RAD-seq data of the *ERV1* inbred F2 population provided us the opportunity for detailed analysis of the genomic condition of each F2 line. We first analyzed the ratio of homo-SNPs out of total population SNPs for each *ERV1* inbred F2 line. After manual filtering, the genotypes of 135 F2 lines were estimated (**Table S7**). The average proportion of homo-SNPs was 83.49% of the total SNPs, much higher than the theoretical value of 75%; 3.7% (n=5) F2 lines ranged from 63.99%–70%, 26.67% (n=36) ranged from 70%–80%, 49.63% (n=67) ranged from 80%–90%, and 20% (n=27) ranged from 90%–96.02%. The lowest ratio (63.99%) of homo-SNPs was discovered in F2 line 147, and the highest ratio (96.02%) was identified in line 163.

The genomic heterozygosity of each *ERV1* inbred F2 line was then analyzed by comparing the sequencing data with the *Nipponbare* reference genome. After excluding abnormal values, sequencing data of 196 lines out of 216 *ERV1* inbred F2 lines were left for in-depth study (**Table S8**). The mean genomic heterozygosity of the 196 lines was approximately 1.68×10^-4^, of which 30.61% F2 lines (n=60) ranged from 0.16×10^-4^-0.98×10^-4^, 32.14% lines (n=63) ranged from 1.0×10^-4^-1.96×10^-4^, 23.98% lines (n=47) ranged from 2.04×10^-4^-2.99×10^-4^, 11.73% lines (n=23) ranged from 3.00×10^-4^-3.81×10^-4^, and 1.53% lines (n=3) ranged from 4.08×10^-4^-4.57×10^-4^. The lowest genomic heterozygosity (0.16×10^-4^) was identified in line 139, and the highest value (4.57×10^-4^) in line 167.

The spontaneous mutation rate of the *ERV1* inbred F2 lines were further estimated using the comparison results. Only the SNPs that were covered by the sequencing data of over 20 F2 lines were considered as candidate sites of spontaneous mutation (**Table S8**). After a series of filtering and screening, the average spontaneous mutation rate of the *ERV1* inbred F2 lines was approximately 5.94×10^-4^, approximately 3.5-fold higher than the mean genomic heterozygosity of the *ERV1* inbred F2 population, of which 1.53% D2 lines (n=3) ranged from 3.91×10^-4^- 4.98×10^-4^, 55.61% lines (n=109) ranged from 5.05×10^-4^-5.99×10^-4^, 42.35% lines (n=83) ranged from 6.00×10^-4^-6.97×10^-4^, and one line had a mutation rate of 7.01×10^-4^. The lowest spontaneous mutation rate (3.91×10^-4^) was identified in line 4, and the highest value (7.01×10^-4^) in line 30.

## Discussion

A series of previous studies have documented that SSI could induce extensive genomic variations, and result to phenotypic changes of important traits (Zhou et al., 1983; de la Peña et al., 1987; Zhao et al., 2007). They also experimently confirmed the integration of donor DNA fragments in variant genomes (Liu et al., 2000; Miao et al., 2000; Xing et al., 2004), and revealed that allelic variation of important genes might be the main factor for phenotypic changes of variant plants (Wang et al., 2013; Peng et al., 2016). Nevertheless, these studies mainly based on limited molecular markers such as RFLP, RAPD, AFLP and SSR (Shan et al., 2005; Wang et al., 2010), the extent and distribution pattern of the induced variations in a genome-wide scale and its inheritance patterns were largely obscure.

People used to use introgressive hybrid added with embryo rescue to overcome the cross incompatibility between the wild species of genus *Oryza* and cultivated rice, and to generate interspecific hybrids (Ramanujam, 1937; Nayar, 1973). Notably, the rice variant *ERV1* from SSI method seemed to a hybrid F1 cross between *RH78* and *O. eichingeri*, the latter belongs to the wild species of genus *Oryza*, has 2n=24 chromosomes representing CC genome, and is incompatible with *O. sativa* genome (Brar and Khush, 1997). This proved that SSI method could realize the exchange or transfer of genetic materials without the effects of reproductive isolation. Meanwhile, the values of plant height, panicle length, spikelet number, and length and width of flag leaf of the rice variant *ERV1* in this study are all higher than the rice recipient *RH78* but lower than the donor *O. eichingeri*, and the genomic heterozygosity of *ERV1* was approximately 8-fold higher than *RH78*. This phenomenon was similar to the introgressive hybridization by pollens of alien sexually incompatible species (Hongyan et al., 2009; Dowen et al., 2012), which would provoke genome-wide and extensive genomic changes, and even results in important phenotypic novelties (Wang et al., 2013; Sakai et al., 2013; Schiestl and Johnson, 2013). We also noted that the genomic heterozygosity rapidly decreased from as high as 25.96 × 10^-4^ in *ERV1* to an average of approximately 1.68×10^-4^ in *ERV1* inbred F2 lines, this was much different from introgressive hybridization, and indicated that the novel phenotypes or traits of these rice variants generated by SSI method could be quickly fixed, and utilized in crop genetic improvements.

Spontaneous mutation is an important factor in genome evolution and phenotypic variation (Zhang et al., 2016). In this study, we identified a total of 5,518 homozygous SNPs in *ERV1* relative to *RH78*, resulting in a spontaneous mutation rate of 0.182×10^-4^ in *ERV1*. The average spontaneous mutation rate of *ERV1* inbred F2 lines was estimated to be approximately 5.94×10^-4^, significantly increased compared to *ERV1*, and approximately 3.5-fold higher than the mean genomic heterozygosity of the *ERV1* inbred F2 lines. This interesting phenomenon implies a highly un-purified genetic background, and high genetic variability of the *ERV1* genome.

In conclusion, our study presented a method that could quickly and comprehensively improve important agronomic traits of cultivated rice, and preliminarily unveiled the fundamental rules of the genetic variation and inheritance of variation in the early generation *via* multi-omics strategies and comparative genomic analysis. The results enhanced our understanding of SSI methods, and may accelerate our understanding of plant genome evolution, domestication, and speciation in nature.

## Data Availability Statement

The original contributions presented in the study are publicly available. This data can be found here: https://www.ncbi.nlm.nih.gov/bioproject/PRJNA658863.

## Author Contributions

YH, BM, and YX extracted DNA, identified the genotype of F2 population, and wrote the manuscript. YP, DZ and YS performed SNP data analysis and QTL detecting. LT and YL validated some results of the bioinformatics analysis. BZ designed the experiments and supervised the study. All authors contributed to the article and approved the submitted version.

## Funding

This research was financially supported by the Natural Science Foundation of Hunan province (2020JJ5399), the National Major Special Project on New Varieties Cultivation for Transgenic Organisms (Grant No. 2016ZX08001-004), the Major Science and Technology Projects of Hunan province (2018NK1020, 2018NK1010), and the 2020 Research Program of Sanya Yazhou Bay Science and Technology City (Grant No.202002006).

## Conflict of Interest

The authors declare that the research was conducted in the absence of any commercial or financial relationships that could be construed as a potential conflict of interest.
