# Supplementary material for: Spike-Stalk Injection Method Causes Extensive Phenotypic and Genotypic Variations for Rice Germplasm
Source: Front Plant Sci. 2020 Sep 25;11:575373. doi: 10.3389/fpls.2020.575373 (PMC7546333; doi:10.3389/fpls.2020.575373)
Supplement: Supplementary file 1 [file Table_1.docx]

Supplementary Table 1 Comparison of yield -related traits between ERV1 and RH78

|  | Plant Height  (cm) | Tilling Number | Panicle Length  (cm) | Leaf length  (cm) | Leaf width  (cm) | Spikelet Number | Seed Setting Rate  (%) | 100-grains weight  (g) |
| --- | --- | --- | --- | --- | --- | --- | --- | --- |
| ERV1 | 131.2 | 11 | 28.7 | 48.1 | 2.8 | 325 | 83 | 3.13 |
| RH78 | 99.8±6.72 | 10.8±1.3 | 26.1±1.24 | 40.9±3.55 | 2±0.15 | 201.9±16.8 | 75.1±2.33 | 3.08±0.11 |
| P value | 1.65474E-11 | 0.63674044 | 3.75522E-06 | 4.83909E-06 | 2.88944E-12 | 7.25035E-15 | 3.04297E-09 | 0.197430451 |
