# Supplementary material for: Spike-Stalk Injection Method Causes Extensive Phenotypic and Genotypic Variations for Rice Germplasm
Source: Front Plant Sci. 2020 Sep 25;11:575373. doi: 10.3389/fpls.2020.575373 (PMC7546333; doi:10.3389/fpls.2020.575373)
Supplement: Supplementary file 2 [file Table_2.docx]

Supplementary Table 2 Statistics of SNPs in RH78 relative to Nipponbare genome

| Chr | Reference  Size(bp) | covered bases | Sequencing Coverage  (%) | Sequencing Depth  (fold) | Total  SNPs Number | Total SNP density | Homo-  SNPs number | Homo-  SNPs density | Hetero-  SNPs number | Hetero-  SNPs density | Hetero-SNPs  Ratio(%) | Covered-region  Heterozygosity  （10^-4^） | Genomic  Heterozygosity（10^-4^） |
| --- | --- | --- | --- | --- | --- | --- | --- | --- | --- | --- | --- | --- | --- |
| chr01 | 43,268,879 | 35,918,817 | 83.01 | 26.53 | 166,664 | 4.64 | 153,512 | 4.27 | 9,739 | 0.27 | 5.84 | 2.71 | 2.25 |
| chr02 | 35,930,381 | 30,241,173 | 84.17 | 27.22 | 148,411 | 4.91 | 136,002 | 4.50 | 9,086 | 0.30 | 6.12 | 3.00 | 2.53 |
| chr03 | 36,406,689 | 32,031,725 | 87.98 | 28.84 | 145,748 | 4.55 | 133,144 | 4.16 | 9,357 | 0.29 | 6.42 | 2.92 | 2.57 |
| chr04 | 35,278,225 | 28,130,555 | 79.74 | 26.12 | 110,696 | 3.94 | 97,252 | 3.46 | 9,618 | 0.34 | 8.69 | 3.42 | 2.73 |
| chr05 | 29,894,789 | 25,895,781 | 86.62 | 28.23 | 96,711 | 3.73 | 85,596 | 3.31 | 8,062 | 0.31 | 8.34 | 3.11 | 2.70 |
| chr06 | 31,246,789 | 25,694,017 | 82.23 | 27.96 | 107,308 | 4.18 | 95,118 | 3.70 | 8,965 | 0.35 | 8.35 | 3.49 | 2.87 |
| chr07 | 29,696,629 | 23,304,030 | 78.47 | 26.02 | 113,447 | 4.87 | 102,463 | 4.40 | 8,009 | 0.34 | 7.06 | 3.44 | 2.70 |
| chr08 | 28,439,308 | 23,163,124 | 81.45 | 27.32 | 107,410 | 4.64 | 94,802 | 4.09 | 8,874 | 0.38 | 8,26 | 3.83 | 3.12 |
| chr09 | 23,011,239 | 18,373,571 | 79.85 | 26.13 | 94,239 | 5.13 | 84,920 | 4.62 | 6,791 | 0.37 | 7.21 | 3.70 | 2.95 |
| chr10 | 23,134,759 | 18,267,662 | 78.96 | 27.03 | 97,103 | 5.32 | 87,938 | 4.81 | 6,722 | 0.37 | 6.92 | 3.68 | 2.91 |
| chr11 | 28,512,666 | 21,293,103 | 74.68 | 25.58 | 120,987 | 5.68 | 108,557 | 5.10 | 8,839 | 0.42 | 7.31 | 4.15 | 3.10 |
| chr12 | 27,497,214 | 20,769,044 | 75.53 | 28.64 | 88,911 | 4.28 | 78,293 | 3.77 | 7,760 | 0.37 | 8.73 | 3.74 | 2.82 |
| Total/Average | 372,317,567 | 303,082,602 | 81.06 | 27.14 | 1,397,635 | 4.65 | 1,257,597 | 4.18 | 101,822 | 0.34 | 7.29 | 3.36 | 2.73 |
