# Supplementary material for: Spike-Stalk Injection Method Causes Extensive Phenotypic and Genotypic Variations for Rice Germplasm
Source: Front Plant Sci. 2020 Sep 25;11:575373. doi: 10.3389/fpls.2020.575373 (PMC7546333; doi:10.3389/fpls.2020.575373)
Supplement: Supplementary file 3 [file Table_3.docx]

Supplementary Table 3 Statistics of SNPs in ERV1 relative to Nipponbare genome

| Chr | Reference  Size(bp) | covered bases | Sequencing Coverage  (%) | Sequencing Depth  (fold) | Total  SNPs | Total SNP density | Homo-  SNPs | Homo-  SNPs density | Hetero-  SNPs | Hetero-  SNPs density | Hetero-  SNPs  Ratio(%) | Covered-region  Heterozygosity  （10^-4^） | Genomic  Heterozygosity（10^-4^） |
| --- | --- | --- | --- | --- | --- | --- | --- | --- | --- | --- | --- | --- | --- |
| chr01 | 43,268,879 | 36,552,437 | 84.48 | 29.14 | 219,370 | 6.00 | 144,925 | 3.96 | 74,445 | 2.04 | 33.94 | 20.37 | 17.21 |
| chr02 | 35,930,381 | 31,027,682 | 86.36 | 29.57 | 187,026 | 6.03 | 107,850 | 3.48 | 79,176 | 2.55 | 42.33 | 25.52 | 22.04 |
| chr03 | 36,406,689 | 32,673,735 | 89.75 | 30.01 | 190,474 | 5.83 | 116,735 | 3.57 | 73,739 | 2.26 | 38.71 | 22.57 | 20.25 |
| chr04 | 35,278,225 | 29,150,685 | 82.63 | 30.35 | 147,889 | 5.07 | 67,403 | 2.31 | 80,486 | 2.76 | 54.42 | 27.61 | 22.81 |
| chr05 | 29,894,789 | 26,433,643 | 88.42 | 30.26 | 146,866 | 5.56 | 67,663 | 2.56 | 79,203 | 3.00 | 53.93 | 29.96 | 26.49 |
| chr06 | 31,246,789 | 26,733,438 | 85.56 | 29.88 | 158,251 | 5.92 | 75,260 | 2.82 | 82,991 | 3.10 | 52.44 | 31.04 | 26.56 |
| chr07 | 29,696,629 | 23,992,042 | 80.79 | 27.36 | 149,983 | 6.25 | 101,815 | 4.24 | 48,168 | 2.01 | 32.12 | 20.08 | 16.22 |
| chr08 | 28,439,308 | 23,655,381 | 83.18 | 28.47 | 146,706 | 6.20 | 90,809 | 3.84 | 55,897 | 2.36 | 38.10 | 23.63 | 19.65 |
| chr09 | 23,011,239 | 19,191,193 | 83.40 | 29.36 | 122,420 | 6.38 | 62,849 | 3.27 | 59,571 | 3.10 | 48.66 | 31.04 | 25.89 |
| chr10 | 23,134,759 | 19,042,670 | 82.31 | 27.55 | 138,066 | 7.25 | 82,845 | 4.35 | 55,221 | 2.90 | 40.00 | 29.00 | 23.87 |
| chr11 | 28,512,666 | 21,786,265 | 76.41 | 27.13 | 152,435 | 7.00 | 109,101 | 5.01 | 43,334 | 1.99 | 28.43 | 19.90 | 15.20 |
| chr12 | 27,497,214 | 22,158,159 | 80.58 | 29.27 | 139,020 | 6.27 | 60,277 | 2.72 | 78,743 | 3.55 | 56.64 | 35.54 | 28.64 |
| Total/Average | 372,317,567 | 312,397,330 | 83.66 | 29.03 | 1,898,506 | 6.15 | 1,087,532 | 3.51 | 810,974 | 2.64 | 42.72 | 25.96 | 21.78 |
