# Supplementary material for: Spike-Stalk Injection Method Causes Extensive Phenotypic and Genotypic Variations for Rice Germplasm
Source: Front Plant Sci. 2020 Sep 25;11:575373. doi: 10.3389/fpls.2020.575373 (PMC7546333; doi:10.3389/fpls.2020.575373)
Supplement: Supplementary file 4 [file Table_4.docx]

Supplementary Table 4 Annotation of hetero-SNPs in ERV1 relative to RH78

| chr | mRNA | exon | CDS | 5_UTR | 3_UTR |
| --- | --- | --- | --- | --- | --- |
| Chr1 | 7228 | 19021 | 15951 | 2388 | 3414 |
| Chr2 | 5969 | 15797 | 13115 | 1943 | 2904 |
| Chr3 | 6402 | 17245 | 14470 | 2269 | 3021 |
| Chr4 | 5073 | 12071 | 10572 | 1232 | 1728 |
| Chr5 | 4773 | 12012 | 10296 | 1349 | 1958 |
| Chr6 | 4474 | 10930 | 9561 | 1073 | 1595 |
| Chr7 | 4540 | 11446 | 9919 | 1217 | 1826 |
| Chr8 | 4263 | 11094 | 9646 | 1149 | 1583 |
| Chr9 | 3515 | 8972 | 7811 | 921 | 1327 |
| Chr10 | 3611 | 9652 | 8470 | 1049 | 1298 |
| Chr11 | 3945 | 10214 | 9168 | 873 | 1244 |
| Chr12 | 3876 | 9669 | 8565 | 977 | 1212 |
| Total | 57669 | 148123 | 127544 | 16440 | 23110 |
