# Supplementary material for: Spike-Stalk Injection Method Causes Extensive Phenotypic and Genotypic Variations for Rice Germplasm
Source: Front Plant Sci. 2020 Sep 25;11:575373. doi: 10.3389/fpls.2020.575373 (PMC7546333; doi:10.3389/fpls.2020.575373)
Supplement: Supplementary file 5 [file Table_5.docx]

Supplementary Table 5 Statistics of sequencing information of ERV1 inbred F2 lines

| Sample | Total reads(M) | Total base(M) | Mapped reads(M) | Mapped base(M) |
| --- | --- | --- | --- | --- |
| 1 | 1.407762 | 126.69858 | 1.12884 | 101.5956 |
| 2 | 2.457632 | 221.18688 | 1.970689 | 177.36201 |
| 3 | 0.143548 | 12.91932 | 0.110562 | 9.95058 |
| 11 | 0.18598 | 16.7382 | 0.142489 | 12.82401 |
| 16 | 1.164718 | 104.82462 | 0.892673 | 80.34057 |
| 18 | 1.138526 | 102.46734 | 0.926539 | 83.38851 |
| 20 | 2.343668 | 210.93012 | 1.904794 | 171.43146 |
| 22 | 2.35245 | 211.7205 | 1.882064 | 169.38576 |
| 23 | 2.297684 | 206.79156 | 1.813064 | 163.17576 |
| 28 | 0.433964 | 39.05676 | 0.353982 | 31.85838 |
| 33 | 2.098984 | 188.90856 | 1.676912 | 150.92208 |
| 35 | 2.502894 | 225.26046 | 1.960803 | 176.47227 |
| 40 | 1.878626 | 169.07634 | 1.500413 | 135.03717 |
| 43 | 1.083366 | 97.50294 | 0.853752 | 76.83768 |
| 50 | 0.874514 | 78.70626 | 0.68341 | 61.5069 |
| 51 | 1.231534 | 110.83806 | 0.908259 | 81.74331 |
| 52 | 1.156396 | 104.07564 | 0.91741 | 82.5669 |
| 54 | 2.049076 | 184.41684 | 1.645008 | 148.05072 |
| 55 | 0.65067 | 58.5603 | 0.528151 | 47.53359 |
| 56 | 2.077092 | 186.93828 | 1.680549 | 151.24941 |
| 58 | 0.707032 | 63.63288 | 0.539224 | 48.53016 |
| 61 | 0.605056 | 54.45504 | 0.485968 | 43.73712 |
| 64 | 2.95932 | 266.3388 | 2.276874 | 204.91866 |
| 66 | 2.192862 | 197.35758 | 1.758979 | 158.30811 |
| 67 | 2.555964 | 230.03676 | 2.064187 | 185.77683 |
| 69 | 1.865698 | 167.91282 | 1.422412 | 128.01708 |
| 76 | 0.615294 | 55.37646 | 0.460031 | 41.40279 |
| 82 | 0.742274 | 66.80466 | 0.56932 | 51.2388 |
| 85 | 2.418802 | 217.69218 | 1.732034 | 155.88306 |
| 86 | 0.592482 | 53.32338 | 0.445419 | 40.08771 |
| 91 | 0.516526 | 46.48734 | 0.409536 | 36.85824 |
| 92 | 0.440982 | 39.68838 | 0.353081 | 31.77729 |
| 93 | 1.417104 | 127.53936 | 1.117256 | 100.55304 |
| 95 | 1.260668 | 113.46012 | 1.015279 | 91.37511 |
| 99 | 2.017486 | 181.57374 | 1.636024 | 147.24216 |
| 102 | 2.14339 | 192.9051 | 1.626445 | 146.38005 |
| 104 | 1.544272 | 138.98448 | 1.221553 | 109.93977 |
| 106 | 1.348144 | 121.33296 | 1.035142 | 93.16278 |
| 114 | 1.103292 | 99.29628 | 0.866558 | 77.99022 |
| 116 | 1.212704 | 109.14336 | 0.830371 | 74.73339 |
| 119 | 0.100392 | 9.03528 | 0.081171 | 7.30539 |
| 121 | 0.998714 | 89.88426 | 0.753057 | 67.77513 |
| 124 | 2.13676 | 192.3084 | 1.580613 | 142.25517 |
| 127 | 0.85967 | 77.3703 | 0.609673 | 54.87057 |
| 130 | 0.80765 | 72.6885 | 0.601262 | 54.11358 |
| 137 | 0.93187 | 83.8683 | 0.724838 | 65.23542 |
| 140 | 2.75593 | 248.0337 | 2.208525 | 198.76725 |
| 146 | 3.076986 | 276.92874 | 2.481838 | 223.36542 |
| 150 | 2.377788 | 214.00092 | 1.790078 | 161.10702 |
| 152 | 4.435258 | 399.17322 | 3.539114 | 318.52026 |
| 155 | 0.096424 | 8.67816 | 0.075468 | 6.79212 |
| 156 | 1.830508 | 164.74572 | 1.455517 | 130.99653 |
| 157 | 0.740622 | 66.65598 | 0.591976 | 53.27784 |
| 167 | 1.846822 | 166.21398 | 1.487215 | 133.84935 |
| 168 | 0.084028 | 7.56252 | 0.063327 | 5.69943 |
| 171 | 3.230018 | 290.70162 | 2.449141 | 220.42269 |
| 174 | 0.957904 | 86.21136 | 0.731515 | 65.83635 |
| 176 | 4.067852 | 366.10668 | 2.911344 | 262.02096 |
| 187 | 0.97323 | 87.5907 | 0.761321 | 68.51889 |
| 194 | 3.369782 | 303.28038 | 2.663636 | 239.72724 |
| 196 | 0.832648 | 74.93832 | 0.65596 | 59.0364 |
| 197 | 3.234638 | 291.11742 | 2.492485 | 224.32365 |
| 198 | 0.546844 | 49.21596 | 0.426625 | 38.39625 |
| 201 | 1.389732 | 125.07588 | 1.095611 | 98.60499 |
| 205 | 2.067912 | 186.11208 | 1.613059 | 145.17531 |
| 209 | 1.335536 | 120.19824 | 1.058277 | 95.24493 |
| 224 | 2.8612 | 257.508 | 2.311373 | 208.02357 |
| 228 | 3.40568 | 306.5112 | 2.720011 | 244.80099 |
| 229 | 1.494392 | 134.49528 | 1.20317 | 108.2853 |
| 230 | 2.44905 | 220.4145 | 1.980514 | 178.24626 |
| 236 | 1.685684 | 151.71156 | 1.362178 | 122.59602 |
| 239 | 0.295922 | 26.63298 | 0.231557 | 20.84013 |
| 243 | 0.322192 | 28.99728 | 0.259618 | 23.36562 |
| 244 | 0.228528 | 20.56752 | 0.177144 | 15.94296 |
| 250 | 1.192018 | 107.28162 | 0.968589 | 87.17301 |
| 251 | 0.555082 | 49.95738 | 0.446431 | 40.17879 |
| 252 | 1.929806 | 173.68254 | 1.548659 | 139.37931 |
| 255 | 1.241734 | 111.75606 | 0.994136 | 89.47224 |
| 258 | 0.504698 | 45.42282 | 0.410824 | 36.97416 |
| 261 | 4.62441 | 416.1969 | 3.736769 | 336.30921 |
| 265 | 3.401996 | 306.17964 | 2.761429 | 248.52861 |
| 266 | 2.27178 | 204.4602 | 1.813503 | 163.21527 |
| 267 | 2.108038 | 189.72342 | 1.668067 | 150.12603 |
| 268 | 2.576304 | 231.86736 | 2.078874 | 187.09866 |
| 270 | 0.918492 | 82.66428 | 0.739998 | 66.59982 |
| 272 | 0.889114 | 80.02026 | 0.706697 | 63.60273 |
| 274 | 9.733724 | 876.03516 | 7.794714 | 701.52426 |
| 275 | 2.769036 | 249.21324 | 2.116348 | 190.47132 |
| 276 | 0.335208 | 30.16872 | 0.266191 | 23.95719 |
| 277 | 0.469922 | 42.29298 | 0.379736 | 34.17624 |
| 278 | 0.04811 | 4.3299 | 0.020224 | 1.82016 |
| 279 | 2.213192 | 199.18728 | 1.76261 | 158.6349 |
| 281 | 0.746114 | 67.15026 | 0.596013 | 53.64117 |
| 285 | 0.7064 | 63.576 | 0.443044 | 39.87396 |
| 296 | 2.296638 | 206.69742 | 1.860711 | 167.46399 |
| 298 | 1.812544 | 163.12896 | 1.431055 | 128.79495 |
| 299 | 3.384002 | 304.56018 | 2.721715 | 244.95435 |
| 301 | 1.896078 | 170.64702 | 1.503418 | 135.30762 |
| 303 | 1.577706 | 141.99354 | 1.144041 | 102.96369 |
| 309 | 1.906822 | 171.61398 | 1.504778 | 135.43002 |
| 310 | 0.377316 | 33.95844 | 0.289641 | 26.06769 |
| 311 | 0.986984 | 88.82856 | 0.786113 | 70.75017 |
| 318 | 2.113402 | 190.20618 | 1.662679 | 149.64111 |
| 327 | 1.87776 | 168.9984 | 1.509817 | 135.88353 |
| 328 | 2.347276 | 211.25484 | 1.777291 | 159.95619 |
| 331 | 1.304008 | 117.36072 | 0.978311 | 88.04799 |
| 332 | 1.201064 | 108.09576 | 0.949449 | 85.45041 |
| 336 | 3.559694 | 320.37246 | 2.771674 | 249.45066 |
| 339 | 2.657638 | 239.18742 | 2.043534 | 183.91806 |
| 343 | 1.745672 | 157.11048 | 1.314602 | 118.31418 |
| 344 | 0.333182 | 29.98638 | 0.266059 | 23.94531 |
| 345 | 0.964258 | 86.78322 | 0.691224 | 62.21016 |
| 346 | 0.26158 | 23.5422 | 0.20597 | 18.5373 |
| 352 | 2.113238 | 190.19142 | 1.546904 | 139.22136 |
| 358 | 1.722972 | 155.06748 | 1.325349 | 119.28141 |
| 364 | 2.35943 | 212.3487 | 1.693477 | 152.41293 |
| 368 | 1.592074 | 143.28666 | 1.299505 | 116.95545 |
| 372 | 1.991604 | 179.24436 | 1.550229 | 139.52061 |
| 374 | 2.315936 | 208.43424 | 1.806472 | 162.58248 |
| 376 | 1.468466 | 132.16194 | 1.108014 | 99.72126 |
| 380 | 3.021876 | 271.96884 | 2.450736 | 220.56624 |
| 382 | 1.600542 | 144.04878 | 1.274604 | 114.71436 |
| 384 | 5.629014 | 506.61126 | 4.447802 | 400.30218 |
| 385 | 0.998578 | 89.87202 | 0.752565 | 67.73085 |
| 387 | 1.906192 | 171.55728 | 1.36409 | 122.7681 |
| 388 | 1.725072 | 155.25648 | 1.237146 | 111.34314 |
| 389 | 1.722378 | 155.01402 | 1.379373 | 124.14357 |
| 391 | 0.832528 | 74.92752 | 0.674299 | 60.68691 |
| 392 | 0.546978 | 49.22802 | 0.432219 | 38.89971 |
| 393 | 0.906196 | 81.55764 | 0.725365 | 65.28285 |
| 395 | 0.183712 | 16.53408 | 0.14439 | 12.9951 |
| 397 | 2.40648 | 216.5832 | 1.852091 | 166.68819 |
| 398 | 1.376918 | 123.92262 | 1.001674 | 90.15066 |
| 399 | 2.876606 | 258.89454 | 2.220604 | 199.85436 |
| 404 | 1.551092 | 139.59828 | 1.222141 | 109.99269 |
| 405 | 2.30158 | 207.1422 | 1.642309 | 147.80781 |
| 406 | 0.911118 | 82.00062 | 0.716445 | 64.48005 |
| 408 | 2.245856 | 202.12704 | 1.676916 | 150.92244 |
| 410 | 3.807854 | 342.70686 | 2.8659 | 257.931 |
| 413 | 2.127758 | 191.49822 | 1.509763 | 135.87867 |
| 415 | 1.57435 | 141.6915 | 1.222453 | 110.02077 |
| 421 | 0.656974 | 59.12766 | 0.526699 | 47.40291 |
| 422 | 1.078456 | 97.06104 | 0.852413 | 76.71717 |
| 429 | 0.742612 | 66.83508 | 0.593684 | 53.43156 |
| 431 | 1.346348 | 121.17132 | 1.006951 | 90.62559 |
| 440 | 1.171884 | 105.46956 | 0.881495 | 79.33455 |
| 441 | 3.789218 | 341.02962 | 3.070406 | 276.33654 |
| 443 | 3.08676 | 277.8084 | 2.482431 | 223.41879 |
| 444 | 1.320554 | 118.84986 | 1.007615 | 90.68535 |
| 452 | 1.851542 | 166.63878 | 1.493958 | 134.45622 |
| 456 | 1.227402 | 110.46618 | 0.990694 | 89.16246 |
| 459 | 1.117708 | 100.59372 | 0.865755 | 77.91795 |
| 460 | 1.719538 | 154.75842 | 1.350193 | 121.51737 |
| 464 | 0.541596 | 48.74364 | 0.397652 | 35.78868 |
| 467 | 0.577258 | 51.95322 | 0.452082 | 40.68738 |
| 468 | 2.092992 | 188.36928 | 1.697611 | 152.78499 |
| 470 | 1.090544 | 98.14896 | 0.852372 | 76.71348 |
| 471 | 0.454686 | 40.92174 | 0.344004 | 30.96036 |
| 472 | 2.52268 | 227.0412 | 2.04987 | 184.4883 |
| 473 | 2.694824 | 242.53416 | 2.101345 | 189.12105 |
| 475 | 1.7697 | 159.273 | 1.371467 | 123.43203 |
| 477 | 2.766474 | 248.98266 | 1.994011 | 179.46099 |
| 478 | 1.0997 | 98.973 | 0.8779 | 79.011 |
| 479 | 0.833484 | 75.01356 | 0.63102 | 56.7918 |
| 484 | 2.72569 | 245.3121 | 2.148977 | 193.40793 |
| 489 | 1.222932 | 110.06388 | 0.888601 | 79.97409 |
| 492 | 0.434288 | 39.08592 | 0.34853 | 31.3677 |
| 500 | 0.864828 | 77.83452 | 0.692545 | 62.32905 |
| 503 | 0.471152 | 42.40368 | 0.356164 | 32.05476 |
| 505 | 1.826962 | 164.42658 | 1.448569 | 130.37121 |
| 511 | 0.601588 | 54.14292 | 0.401197 | 36.10773 |
| 518 | 1.673352 | 150.60168 | 1.228335 | 110.55015 |
| 520 | 2.201802 | 198.16218 | 1.703485 | 153.31365 |
| 521 | 1.857942 | 167.21478 | 1.485421 | 133.68789 |
| 522 | 1.39508 | 125.5572 | 1.101859 | 99.16731 |
| 528 | 2.129118 | 191.62062 | 1.65019 | 148.5171 |
| 533 | 1.195476 | 107.59284 | 0.920084 | 82.80756 |
| 538 | 1.090596 | 98.15364 | 0.814509 | 73.30581 |
| 541 | 1.805908 | 162.53172 | 1.470183 | 132.31647 |
| 542 | 1.332642 | 119.93778 | 1.070693 | 96.36237 |
| 545 | 2.690624 | 242.15616 | 2.101882 | 189.16938 |
| 547 | 1.61803 | 145.6227 | 1.188255 | 106.94295 |
| 548 | 2.285728 | 205.71552 | 1.8549 | 166.941 |
| 553 | 1.282624 | 115.43616 | 1.013975 | 91.25775 |
| 554 | 0.918302 | 82.64718 | 0.707467 | 63.67203 |
| 555 | 1.48428 | 133.5852 | 1.189994 | 107.09946 |
| 556 | 2.25243 | 202.7187 | 1.718616 | 154.67544 |
| 560 | 1.190976 | 107.18784 | 0.850543 | 76.54887 |
| 561 | 1.145184 | 103.06656 | 0.903551 | 81.31959 |
| 565 | 1.890984 | 170.18856 | 1.52228 | 137.0052 |
| 566 | 3.540742 | 318.66678 | 2.665584 | 239.90256 |
| 567 | 0.79591 | 71.6319 | 0.579506 | 52.15554 |
| 574 | 2.511016 | 225.99144 | 1.97083 | 177.3747 |
| 575 | 0.461368 | 41.52312 | 0.359831 | 32.38479 |
| 577 | 1.01385 | 91.2465 | 0.806097 | 72.54873 |
| 579 | 2.685522 | 241.69698 | 2.137127 | 192.34143 |
| 580 | 3.002326 | 270.20934 | 2.439427 | 219.54843 |
| 581 | 1.84675 | 166.2075 | 1.37206 | 123.4854 |
| 584 | 2.725386 | 245.28474 | 2.177311 | 195.95799 |
| 586 | 2.693096 | 242.37864 | 2.153525 | 193.81725 |
| 587 | 1.220996 | 109.88964 | 0.961199 | 86.50791 |
| 588 | 0.362518 | 32.62662 | 0.283154 | 25.48386 |
| 593 | 0.975094 | 87.75846 | 0.766165 | 68.95485 |
| 596 | 0.98774 | 88.8966 | 0.681382 | 61.32438 |
| 597 | 1.926686 | 173.40174 | 1.49235 | 134.3115 |
| 598 | 1.411506 | 127.03554 | 1.037122 | 93.34098 |
| 605 | 0.01314 | 1.1826 | 0.00961 | 0.8649 |
| 606 | 0.971678 | 87.45102 | 0.783719 | 70.53471 |
| 607 | 0.201672 | 18.15048 | 0.147269 | 13.25421 |
| 610 | 0.030206 | 2.71854 | 0.022803 | 2.05227 |
| 612 | 1.109084 | 99.81756 | 0.835312 | 75.17808 |
| 614 | 0.64489 | 58.0401 | 0.506363 | 45.57267 |
| 615 | 0.20373 | 18.3357 | 0.162494 | 14.62446 |
| 619 | 0.708584 | 63.77256 | 0.571956 | 51.47604 |
| 621 | 1.315388 | 118.38492 | 1.057982 | 95.21838 |
| 629 | 1.24456 | 112.0104 | 0.963169 | 86.68521 |
