# Supplementary material for: Spike-Stalk Injection Method Causes Extensive Phenotypic and Genotypic Variations for Rice Germplasm
Source: Front Plant Sci. 2020 Sep 25;11:575373. doi: 10.3389/fpls.2020.575373 (PMC7546333; doi:10.3389/fpls.2020.575373)
Supplement: Supplementary file 6 [file Table_6.docx]

Supplementary Table 6 Yield-related traits of ERV1 inbred F2 lines

| Sample | Heading stage (day) | Flag leaf length  (cm) | Flag leaf width  (cm) | Plant height  (cm) | Tiller number | panicle length  (cm) | spikelets  Per   panicle | seed setting  rate  (%) | 100-grain  Weight  (g) |
| --- | --- | --- | --- | --- | --- | --- | --- | --- | --- |
| 1 | 109 | 31.5 | 2.3 | 93.8 | 7 | 22.8 | 196 | 79.1% | 3.27 |
| 2 | 107 | 26.3 | 2.6 | 103 | 8 | 21.4 | 201 | 84.6% | 2.6 |
| 3 | 102 | 27.5 | 2.1 | 92.5 | 12 | 21.4 | 216 | 88.0% | 2.43 |
| 4 | 101 | 32 | 2.3 | 93 | 6 | 24.4 | 153 | 96.1% | 3.38 |
| 5 | 105 | 27.5 | 2.6 | 107 | 15 | 23.3 | 235 | 84.3% | 3.21 |
| 6 | 102 | 30 | 2.7 | 97 | 12 | 24.5 | 262 | 72.9% | 2.64 |
| 7 | 103 | 23 | 2.4 | 104 | 9 | 19.8 | 202 | 90.6% | 2.75 |
| 8 | 102 | 24.5 | 2 | 93 | 12 | 21.4 | 148 | 93.9% | 3.42 |
| 9 | 106 | 29 | 2.6 | 97 | 8 | 21.5 | 173 | 85.5% | 3.26 |
| 10 | 107 | 35.5 | 3.1 | 92 | 12 | 21 | 227 | 91.2% | 3.18 |
| 11 | 101 | 31.5 | 2.6 | 95 | 8 | 24 | 186 | 91.4% | 3.22 |
| 12 | 106 | 28 | 2.3 | 105 | 9 | 23.5 | 214 | 69.6% | 3.22 |
| 13 | 104 | 22.7 | 2.1 | 100 | 10 | 20.2 | 156 | 91.0% | 3.34 |
| 14 | 103 | 27.6 | 2.5 | 91.5 | 5 | 22 | 206 | 91.7% | 3.25 |
| 15 | 106 | 32 | 2.9 | 108 | 9 | 22 | 187 | 93.6% | 2.77 |
| 16 | 108 | 38 | 2.2 | 108 | 11 | 23 | 210 | 79.0% | 3.28 |
| 17 | 107 | 25.5 | 2.5 | 100 | 7 | 23.5 | 185 | 88.1% | 2.78 |
| 18 | 106 | 22.8 | 2.3 | 100 | 9 | 20.8 | 222 | 76.1% | 3.36 |
| 19 | 100 | 27.8 | 2.4 | 90 | 9 | 21.5 | 179 | 81.6% | 3.45 |
| 20 | 101 | 26.5 | 2.7 | 96 | 10 | 22 | 285 | 92.6% | 2.51 |
| 21 | 103 | 30.5 | 2.2 | 97 | 10 | 21.5 | 277 | 91.0% | 2.6 |
| 22 | 102 | 27.8 | 2.3 | 100 | 9 | 23 | 181 | 86.7% | 3.26 |
| 23 | 103 | 27.6 | 2.5 | 106 | 17 | 24 | 184 | 93.5% | 3.21 |
| 24 | 104 | 27 | 2.2 | 100 | 10 | 24 | 170 | 95.9% | 3.33 |
| 25 | 103 | 24.3 | 2 | 99.2 | 13 | 23.3 | 191 | 91.6% | 3.24 |
| 26 | 107 | 26.7 | 2.8 | 89 | 6 | 19.5 | 159 | 88.7% | 2.75 |
| 27 | 109 | 28 | 2.4 | 93 | 6 | 23 | 200 | 80.5% | 2.55 |
| 28 | 105 | 26 | 2.9 | 100 | 7 | 21.5 | 222 | 82.0% | 3.21 |
| 29 | 106 | 26 | 2.2 | 105.2 | 12 | 20.8 | 215 | 84.2% | 2.67 |
| 30 | 105 | 23.5 | 2.8 | 93.5 | 8 | 24.1 | 240 | 61.7% | 2.65 |
| 31 | 106 | 27.9 | 2.1 | 104 | 12 | 25.8 | 219 | 84.0% | 3.33 |
| 32 | 106 | 35 | 2.5 | 110 | 8 | 24 | 195 | 83.1% | 3.51 |
| 33 | 109 | 29 | 2.6 | 103 | 8 | 24.3 | 240 | 97.1% | 2.61 |
| 34 | 103 | 26 | 2.1 | 96 | 8 | 24 | 196 | 88.8% | 3.18 |
| 35 | 105 | 28 | 2.1 | 110 | 13 | 23.5 | 235 | 89.4% | 2.63 |
| 36 | 108 | 27 | 2.5 | 101 | 8 | 24 | 238 | 79.4% | 2.68 |
| 37 | 102 | 34 | 2.9 | 94 | 9 | 20.5 | 242 | 95.5% | 2.71 |
| 38 | 106 | 31.6 | 2.4 | 107.5 | 10 | 22.4 | 200 | 92.0% | 3.3 |
| 39 | 95 | 33.8 | 2.6 | 97.5 | 6 | 25.2 | 210 | 59.0% | 3.23 |
| 40 | 103 | 37 | 2.5 | 97 | 6 | 23.8 | 212 | 93.9% | 3.2 |
| 41 | 106 | 28.4 | 2.4 | 101 | 13 | 21.5 | 267 | 91.8% | 2.72 |
| 42 | 104 | 32.5 | 2.8 | 99 | 14 | 21 | 233 | 96.6% | 2.74 |
| 43 | 107 | 29.8 | 2.8 | 106 | 9 | 21.9 | 256 | 94.5% | 2.57 |
| 44 | 106 | 24 | 2.5 | 102 | 11 | 21.5 | 192 | 90.6% | 2.76 |
| 45 | 104 | 22.7 | 2.3 | 97 | 11 | 22.3 | 225 | 96.4% | 2.57 |
| 46 | 109 | 27.5 | 2 | 92 | 10 | 24.5 | 157 | 84.1% | 3.56 |
| 47 | 104 | 26 | 2.2 | 95 | 10 | 23.5 | 175 | 89.7% | 3.25 |
| 48 | 108 | 24.8 | 2.4 | 94.5 | 8 | 21.2 | 280 | 83.2% | 2.62 |
| 49 | 108 | 31.7 | 2.4 | 94 | 11 | 22.8 | 171 | 67.3% | 3.34 |
| 50 | 103 | 26.9 | 2 | 98 | 10 | 22.7 | 188 | 92.0% | 2.72 |
| 51 | 104 | 35.4 | 2.4 | 99 | 12 | 25 | 174 | 97.7% | 3.18 |
| 52 | 108 | 24.5 | 2.3 | 98 | 8 | 22.5 | 180 | 91.1% | 3.21 |
| 53 | 104 | 25 | 2.4 | 95 | 8 | 22 | 201 | 89.6% | 2.72 |
| 54 | 104 | 26.5 | 2.7 | 101 | 9 | 20.3 | 232 | 92.2% | 2.73 |
| 55 | 110 | 26.5 | 2.4 | 110 | 9 | 23.3 | 207 | 75.8% | 3.26 |
| 56 | 114 | 25.3 | 2.3 | 98 | 10 | 21.2 | 289 | 77.9% | 2.48 |
| 57 | 104 | 28.5 | 2.2 | 97 | 9 | 23 | 154 | 96.8% | 3.39 |
| 58 | 102 | 33 | 2.6 | 105 | 8 | 24 | 298 | 95.0% | 2.58 |
| 59 | 102 | 31.6 | 2.5 | 102 | 18 | 23.8 | 261 | 90.0% | 2.59 |
| 60 | 103 | 28 | 2.6 | 110 | 13 | 22.5 | 251 | 92.4% | 2.71 |
| 61 | 103 | 27 | 2.4 | 90 | 7 | 21.5 | 208 | 84.6% | 3.2 |
| 62 | 103 | 25.5 | 2.3 | 95 | 9 | 22 | 222 | 91.0% | 2.62 |
| 63 | 103 | 36 | 2.4 | 95 | 11 | 24 | 203 | 79.3% | 3.3 |
| 64 | 102 | 23 | 2.5 | 92.5 | 5 | 20.5 | 259 | 93.4% | 2.62 |
| 65 | 105 | 24.8 | 2.8 | 105 | 8 | 22.3 | 197 | 88.3% | 2.69 |
| 66 | 109 | 26.5 | 2.8 | 99 | 9 | 23.5 | 173 | 94.2% | 2.74 |
| 67 | 108 | 27 | 2.3 | 97 | 10 | 21 | 129 | 82.2% | 2.55 |
| 68 | 103 | 32 | 2.6 | 100 | 6 | 19.5 | 294 | 87.1% | 2.63 |
| 69 | 109 | 29 | 2.2 | 99 | 4 | 24 | 180 | 87.8% | 3.38 |
| 70 | 107 | 32 | 2.4 | 104 | 14 | 21 | 192 | 89.1% | 3.18 |
| 71 | 105 | 19 | 1.9 | 103 | 8 | 19.3 | 199 | 98.5% | 2.63 |
| 72 | 109 | 32 | 2.1 | 98 | 11 | 24 | 129 | 89.1% | 3.26 |
| 73 | 99 | 22.5 | 1.8 | 100 | 11 | 22 | 200 | 87.5% | 3.24 |
| 74 | 105 | 24.8 | 2.7 | 99.5 | 8 | 22.4 | 217 | 94.5% | 2.64 |
| 75 | 105 | 32 | 2.3 | 106 | 13 | 25.2 | 166 | 96.4% | 3.33 |
| 76 | 110 | 29.5 | 2 | 103 | 10 | 24.5 | 216 | 91.2% | 2.67 |
| 77 | 108 | 21 | 2.2 | 101 | 8 | 23.5 | 160 | 95.0% | 2.69 |
| 78 | 108 | 30.8 | 2.7 | 105 | 12 | 23.5 | 219 | 95.0% | 2.61 |
| 79 | 102 | 32 | 2.5 | 98 | 11 | 25 | 201 | 95.0% | 3.21 |
| 80 | 107 | 25.6 | 2.5 | 90 | 4 | 19 | 156 | 85.3% | 2.56 |
| 81 | 106 | 29 | 2.3 | 104 | 8 | 24.5 | 229 | 78.2% | 3.44 |
| 82 | 106 | 29.5 | 2.8 | 78 | 6 | 19 | 131 | 80.2% | 2.7 |
| 83 | 104 | 32.5 | 2.6 | 93 | 9 | 24.5 | 207 | 78.7% | 3.19 |
| 84 | 102 | 27 | 2 | 93 | 11 | 22.5 | 253 | 93.3% | 2.66 |
| 85 | 103 | 30.5 | 2.4 | 104 | 8 | 24.3 | 162 | 89.5% | 3.38 |
| 86 | 105 | 31 | 2.2 | 102 | 16 | 25.3 | 193 | 94.8% | 2.75 |
| 87 | 104 | 23.6 | 2.5 | 96 | 12 | 21 | 201 | 88.6% | 2.78 |
| 88 | 101 | 29 | 2.4 | 100 | 8 | 20.4 | 193 | 91.2% | 2.78 |
| 89 | 101 | 25.8 | 2.4 | 101 | 12 | 22.5 | 289 | 77.2% | 2.7 |
| 90 | 106 | 30 | 2.4 | 95 | 10 | 22 | 218 | 70.2% | 3.19 |
| 91 | 99 | 24 | 2.1 | 95 | 9 | 25 | 177 | 81.9% | 3.19 |
| 92 | 104 | 24.5 | 2.4 | 109 | 9 | 21.5 | 190 | 95.3% | 2.66 |
| 93 | 104 | 35 | 2.3 | 99 | 8 | 23.4 | 268 | 67.9% | 2.73 |
| 94 | 109 | 21.5 | 2.5 | 91 | 6 | 21.3 | 172 | 67.4% | 2.29 |
| 95 | 107 | 29 | 2.6 | 105 | 10 | 24 | 233 | 93.1% | 2.78 |
| 96 | 109 | 27 | 2 | 96 | 7 | 22 | 161 | 93.8% | 2.75 |
| 97 | 105 | 31 | 2.8 | 98 | 10 | 24.2 | 216 | 81.5% | 3.46 |
| 98 | 105 | 26 | 1.9 | 103 | 10 | 24 | 190 | 85.3% | 3.19 |
| 99 | 106 | 29 | 2.2 | 108 | 12 | 22 | 182 | 97.3% | 3.28 |
| 100 | 102 | 27 | 2.4 | 106 | 11 | 24 | 170 | 84.7% | 3.21 |
| 101 | 104 | 25 | 2.2 | 100 | 11 | 21.8 | 218 | 67.0% | 3.38 |
| 102 | 105 | 37 | 2.2 | 101 | 16 | 21.8 | 194 | 88.1% | 3.32 |
| 103 | 101 | 33 | 2.1 | 96 | 15 | 23.5 | 258 | 92.2% | 2.78 |
| 104 | 107 | 27 | 2.8 | 99 | 13 | 22.2 | 196 | 76.5% | 3.36 |
| 105 | 104 | 25 | 2.1 | 103 | 12 | 23.3 | 208 | 77.4% | 2.63 |
| 106 | 102 | 34 | 2.8 | 100 | 12 | 23 | 286 | 79.7% | 2.65 |
| 107 | 106 | 26 | 2.7 | 100 | 11 | 22.3 | 221 | 95.5% | 2.5 |
| 108 | 105 | 31.6 | 2.3 | 105 | 11 | 25.4 | 254 | 94.1% | 2.72 |
| 109 | 103 | 29 | 2.7 | 99 | 8 | 22 | 262 | 88.5% | 2.54 |
| 110 | 106 | 32 | 2.7 | 94 | 7 | 21 | 159 | 97.5% | 2.77 |
| 111 | 101 | 24 | 2.6 | 94 | 13 | 19 | 245 | 94.3% | 2.78 |
| 112 | 103 | 32 | 2.2 | 94 | 6 | 22.5 | 187 | 86.6% | 3.32 |
| 113 | 99 | 27.5 | 2.3 | 99 | 10 | 20 | 206 | 81.6% | 3.28 |
| 114 | 104 | 25 | 2.2 | 106 | 13 | 22.7 | 281 | 95.4% | 2.72 |
| 115 | 106 | 23.5 | 2.2 | 106 | 11 | 22.3 | 170 | 90.6% | 3.26 |
| 116 | 103 | 21.7 | 2.5 | 92 | 8 | 20.5 | 140 | 93.6% | 2.75 |
| 117 | 103 | 25.5 | 2.3 | 102 | 9 | 20.8 | 235 | 86.0% | 2.73 |
| 118 | 99 | 36.5 | 2.3 | 102 | 14 | 19.7 | 242 | 90.1% | 3.2 |
| 119 | 111 | 26.3 | 2.4 | 90 | 6 | 22 | 211 | 87.7% | 2.46 |
| 120 | 102 | 32 | 2.2 | 92 | 9 | 20.5 | 243 | 89.3% | 2.71 |
| 121 | 109 | 26.5 | 2.1 | 102 | 13 | 21 | 235 | 85.1% | 2.61 |
| 122 | 109 | 29 | 2.2 | 101 | 11 | 23 | 235 | 59.6% | 3.42 |
| 123 | 102 | 24.5 | 2.4 | 99 | 12 | 23 | 234 | 92.7% | 2.76 |
| 124 | 108 | 23 | 2.5 | 90 | 7 | 20.5 | 166 | 78.3% | 2.35 |
| 125 | 104 | 30.7 | 2.3 | 102 | 8 | 24.2 | 157 | 94.3% | 3.28 |
| 126 | 101 | 22 | 2.5 | 94.5 | 7 | 20.5 | 204 | 66.2% | 3.28 |
| 127 | 100 | 26.5 | 1.9 | 100 | 17 | 22.2 | 196 | 80.1% | 3.28 |
| 128 | 109 | 25 | 2.6 | 96 | 7 | 20.6 | 229 | 83.0% | 2.62 |
| 129 | 107 | 24 | 2.7 | 92 | 9 | 20 | 156 | 76.9% | 3.22 |
| 130 | 103 | 34 | 2.7 | 105 | 7 | 23.2 | 285 | 90.5% | 2.74 |
| 131 | 108 | 25 | 2.5 | 97 | 8 | 22 | 201 | 82.1% | 2.64 |
| 132 | 104 | 30.5 | 2.7 | 104 | 9 | 22.8 | 251 | 76.5% | 3.37 |
| 133 | 99 | 29.5 | 2.4 | 87 | 5 | 22.5 | 198 | 84.8% | 3.41 |
| 134 | 104 | 22 | 2.3 | 100 | 13 | 23.5 | 259 | 91.9% | 2.65 |
| 135 | 104 | 32 | 2.5 | 97 | 13 | 25 | 181 | 95.0% | 3.24 |
| 136 | 105 | 38 | 2.4 | 105 | 9 | 22.2 | 174 | 87.9% | 3.35 |
| 137 | 104 | 26.5 | 1.9 | 102 | 10 | 23 | 171 | 86.5% | 3.45 |
| 138 | 103 | 25.5 | 2.5 | 98 | 10 | 22 | 167 | 90.4% | 2.77 |
| 139 | 105 | 35 | 2.3 | 104 | 12 | 25.5 | 219 | 88.6% | 3.24 |
| 140 | 109 | 29 | 2.8 | 100 | 9 | 23.5 | 230 | 79.1% | 2.65 |
| 141 | 105 | 38 | 2.8 | 92 | 11 | 22 | 228 | 82.9% | 2.75 |
| 142 | 105 | 29 | 2.4 | 104 | 9 | 23 | 174 | 94.8% | 3.56 |
| 143 | 108 | 25 | 2.6 | 102 | 12 | 21.7 | 198 | 86.9% | 2.65 |
| 144 | 102 | 32.5 | 2.6 | 87 | 8 | 22.8 | 257 | 72.4% | 2.6 |
| 145 | 107 | 27 | 2.4 | 97 | 9 | 21.5 | 193 | 73.1% | 3.25 |
| 146 | 106 | 30 | 2.5 | 107 | 10 | 22 | 234 | 87.2% | 2.71 |
| 147 | 104 | 27.3 | 2.5 | 100 | 9 | 23.5 | 226 | 96.9% | 2.72 |
| 148 | 105 | 34.5 | 2.4 | 99 | 10 | 24.5 | 182 | 92.9% | 3.22 |
| 149 | 104 | 38 | 2.1 | 106 | 11 | 24.5 | 225 | 67.1% | 3.27 |
| 150 | 107 | 25 | 2 | 99 | 12 | 21.5 | 154 | 83.1% | 3.5 |
| 151 | 106 | 28.5 | 2 | 99 | 6 | 21 | 120 | 82.5% | 3.2 |
| 152 | 107 | 27.5 | 2.7 | 101 | 10 | 23 | 153 | 83.7% | 3.18 |
| 153 | 102 | 29 | 2.4 | 107 | 17 | 23.7 | 258 | 93.4% | 2.61 |
| 154 | 104 | 30 | 2 | 98 | 11 | 24 | 193 | 89.6% | 3.23 |
| 155 | 110 | 35.4 | 2.8 | 98.5 | 11 | 19.4 | 267 | 97.0% | 2.75 |
| 156 | 103 | 32 | 2.5 | 99 | 7 | 23.5 | 191 | 91.6% | 3.34 |
| 157 | 105 | 32 | 2.5 | 100 | 17 | 23.5 | 194 | 87.1% | 3.55 |
| 158 | 105 | 22 | 2.5 | 90 | 6 | 21.5 | 215 | 73.5% | 2.49 |
| 159 | 104 | 27 | 2.1 | 90 | 11 | 21.4 | 157 | 80.9% | 3.38 |
| 160 | 106 | 21 | 2.4 | 105 | 9 | 20.5 | 187 | 68.4% | 3.2 |
| 161 | 102 | 36.5 | 2.3 | 98 | 10 | 22.5 | 215 | 93.5% | 3.2 |
| 162 | 106 | 33 | 2.2 | 100 | 10 | 25 | 180 | 89.4% | 3.76 |
| 163 | 106 | 25.6 | 2.6 | 110 | 13 | 23 | 240 | 85.0% | 2.77 |
| 164 | 107 | 26.5 | 2.5 | 101 | 11 | 21.6 | 183 | 90.2% | 3.36 |
| 165 | 111 | 27.5 | 2.2 | 98 | 7 | 22 | 155 | 93.5% | 2.51 |
| 166 | 108 | 25.5 | 2.5 | 103 | 13 | 22 | 201 | 65.2% | 2.63 |
| 167 | 95 | 25 | 2.4 | 91 | 11 | 20.5 | 241 | 89.6% | 2.7 |
| 168 | 109 | 26.5 | 2.4 | 101 | 9 | 21 | 238 | 91.2% | 2.59 |
| 169 | 99 | 27 | 2.2 | 94 | 8 | 21 | 253 | 88.5% | 3.26 |
| 170 | 107 | 24.8 | 2.3 | 104.5 | 10 | 22.4 | 207 | 72.9% | 2.68 |
| 171 | 107 | 24 | 2.6 | 98 | 7 | 19.5 | 198 | 77.3% | 2.68 |
| 172 | 102 | 36.5 | 2.1 | 96 | 14 | 25.3 | 203 | 86.7% | 3.34 |
| 173 | 103 | 28 | 2.5 | 97 | 7 | 24.8 | 220 | 88.6% | 2.72 |
| 174 | 104 | 23.5 | 2.1 | 104 | 9 | 22 | 138 | 87.7% | 3.24 |
| 175 | 97 | 25.5 | 2.2 | 91 | 10 | 22.8 | 295 | 77.3% | 3.2 |
| 176 | 107 | 28 | 2.6 | 95 | 7 | 20.8 | 165 | 80.6% | 3.25 |
| 177 | 105 | 32.5 | 2.8 | 95 | 12 | 24 | 270 | 77.8% | 3.19 |
| 178 | 100 | 23.5 | 2.6 | 106 | 9 | 20.5 | 214 | 93.0% | 2.71 |
| 179 | 104 | 26.5 | 2.6 | 95 | 8 | 20 | 218 | 82.1% | 2.6 |
| 180 | 101 | 32.5 | 2.4 | 105 | 9 | 23.5 | 282 | 75.2% | 2.66 |
| 181 | 100 | 26.8 | 2 | 104 | 14 | 23.7 | 264 | 74.6% | 2.71 |
| 182 | 102 | 33 | 2.5 | 113 | 13 | 20.5 | 199 | 94.0% | 2.74 |
| 183 | 106 | 27 | 2.2 | 87 | 4 | 21 | 130 | 80.8% | 3.54 |
| 184 | 102 | 27 | 2.1 | 96 | 12 | 22.2 | 249 | 69.5% | 2.74 |
| 185 | 108 | 23.5 | 2.5 | 99 | 8 | 21.5 | 228 | 96.5% | 2.54 |
| 186 | 99 | 26.3 | 1.9 | 92 | 11 | 20.5 | 204 | 85.3% | 3.22 |
| 187 | 103 | 21 | 2.2 | 106 | 12 | 21 | 233 | 93.6% | 2.66 |
| 188 | 103 | 24 | 2.3 | 98 | 7 | 23 | 240 | 78.3% | 3.18 |
| 189 | 99 | 30.5 | 1.9 | 97 | 11 | 22 | 276 | 82.6% | 3.21 |
| 190 | 102 | 26 | 1.9 | 107 | 13 | 22 | 165 | 89.7% | 3.43 |
| 191 | 104 | 36 | 2.2 | 101 | 9 | 21.5 | 155 | 91.0% | 3.43 |
| 192 | 105 | 36.5 | 2.4 | 104 | 8 | 19.5 | 182 | 95.1% | 2.67 |
| 193 | 112 | 33 | 2.2 | 94 | 9 | 23 | 182 | 69.2% | 3.3 |
| 194 | 101 | 28.5 | 2.1 | 90 | 9 | 21.5 | 226 | 85.4% | 3.3 |
| 195 | 100 | 27.8 | 2.3 | 97 | 13 | 20.5 | 224 | 96.9% | 2.68 |
| 196 | 103 | 23.6 | 2.6 | 91.5 | 4 | 21.2 | 166 | 88.6% | 3.2 |
| 197 | 104 | 35 | 2.6 | 92 | 12 | 21 | 209 | 85.6% | 2.75 |
| 198 | 99 | 29 | 2.6 | 86.5 | 6 | 22.4 | 238 | 92.4% | 2.7 |
| 199 | 104 | 27.5 | 1.9 | 106 | 11 | 21.5 | 176 | 97.7% | 2.55 |
| 200 | 106 | 26.3 | 2.5 | 99 | 8 | 22.5 | 173 | 90.2% | 3.37 |
| 201 | 100 | 28.5 | 2.1 | 89 | 8 | 22 | 203 | 88.7% | 3.2 |
| 202 | 103 | 23 | 2.5 | 104 | 7 | 23.5 | 276 | 77.5% | 2.5 |
| 203 | 108 | 26 | 2.4 | 104 | 10 | 23.5 | 174 | 81.6% | 3.57 |
| 204 | 100 | 31 | 2.2 | 95 | 8 | 24.5 | 214 | 89.7% | 3.29 |
| 205 | 105 | 22 | 2 | 101 | 10 | 20.5 | 185 | 88.6% | 3.45 |
| 206 | 105 | 27.5 | 2 | 113 | 18 | 21.3 | 198 | 88.9% | 3.25 |
| 207 | 102 | 33 | 2.4 | 105 | 13 | 24.3 | 252 | 90.9% | 2.68 |
| 208 | 104 | 35.5 | 2.2 | 102 | 16 | 23.5 | 237 | 72.6% | 3.41 |
| 209 | 107 | 31.5 | 2.5 | 110 | 11 | 23.5 | 202 | 88.6% | 2.64 |
| 210 | 103 | 33 | 2.2 | 105 | 13 | 25.5 | 199 | 95.5% | 3.41 |
| 211 | 103 | 26.5 | 2.4 | 95 | 10 | 22.5 | 251 | 92.4% | 2.61 |
| 212 | 105 | 27 | 2.2 | 115 | 14 | 23 | 234 | 86.3% | 3.19 |
| 213 | 108 | 34.7 | 2.5 | 97 | 8 | 24.3 | 253 | 68.8% | 3.23 |
| 214 | 108 | 26 | 2.4 | 105 | 13 | 22 | 229 | 75.1% | 2.73 |
| 215 | 107 | 34.7 | 2 | 96 | 13 | 21.5 | 227 | 56.8% | 3.34 |
| 216 | 105 | 25.7 | 2.3 | 105 | 11 | 22.8 | 204 | 82.4% | 3.35 |
