# Supplementary material for: Spike-Stalk Injection Method Causes Extensive Phenotypic and Genotypic Variations for Rice Germplasm
Source: Front Plant Sci. 2020 Sep 25;11:575373. doi: 10.3389/fpls.2020.575373 (PMC7546333; doi:10.3389/fpls.2020.575373)
Supplement: Supplementary file 7 [file Table_7.docx]

Supplementary Table 7 Statistics of genotype of ERV1 inbred F2 lines

| Sample | Population  SNP | Covered  sites | Homo  -SNPs | Hete  -SNPs | Mutation  site | Miss data | ratio of homo-SNPs |
| --- | --- | --- | --- | --- | --- | --- | --- |
| 1 | 29531 | 23062 | 21442 | 1599 | 21 | 6469 | 92.98% |
| 2 | 29531 | 26191 | 21422 | 4762 | 7 | 3340 | 81.79% |
| 7 | 29531 | 21392 | 17138 | 4241 | 13 | 8139 | 80.11% |
| 8 | 29531 | 25323 | 23230 | 2080 | 13 | 4208 | 91.73% |
| 9 | 29531 | 25273 | 18414 | 6851 | 8 | 4258 | 72.86% |
| 11 | 29531 | 25031 | 19799 | 5219 | 13 | 4500 | 79.10% |
| 12 | 29531 | 24991 | 21071 | 3913 | 7 | 4540 | 84.31% |
| 13 | 29531 | 25072 | 21011 | 4046 | 15 | 4459 | 83.80% |
| 16 | 29531 | 21302 | 19943 | 1343 | 16 | 8229 | 93.62% |
| 18 | 29531 | 23709 | 18282 | 5414 | 13 | 5822 | 77.11% |
| 20 | 29531 | 24607 | 20959 | 3638 | 10 | 4924 | 85.17% |
| 23 | 29531 | 25523 | 19840 | 5670 | 13 | 4008 | 77.73% |
| 24 | 29531 | 24493 | 20713 | 3770 | 10 | 5038 | 84.57% |
| 25 | 29531 | 24744 | 20408 | 4327 | 9 | 4787 | 82.48% |
| 26 | 29531 | 23459 | 21037 | 2408 | 14 | 6072 | 89.68% |
| 29 | 29531 | 25783 | 20969 | 4800 | 14 | 3748 | 81.33% |
| 33 | 29531 | 22802 | 20319 | 2470 | 13 | 6729 | 89.11% |
| 35 | 29531 | 24536 | 20697 | 3833 | 6 | 4995 | 84.35% |
| 36 | 29531 | 23655 | 19398 | 4242 | 15 | 5876 | 82.00% |
| 37 | 29531 | 23995 | 20989 | 2992 | 14 | 5536 | 87.47% |
| 38 | 29531 | 21669 | 19330 | 2333 | 6 | 7862 | 89.21% |
| 40 | 29531 | 20988 | 19922 | 1049 | 17 | 8543 | 94.92% |
| 43 | 29531 | 24875 | 21713 | 3143 | 19 | 4656 | 87.29% |
| 47 | 29531 | 25531 | 19315 | 6206 | 10 | 4000 | 75.65% |
| 48 | 29531 | 26537 | 19155 | 7370 | 12 | 2994 | 72.18% |
| 49 | 29531 | 25845 | 20779 | 5059 | 7 | 3686 | 80.40% |
| 50 | 29531 | 28033 | 21320 | 6706 | 7 | 1498 | 76.05% |
| 52 | 29531 | 23984 | 19281 | 4686 | 17 | 5547 | 80.39% |
| 54 | 29531 | 23284 | 19915 | 3353 | 16 | 6247 | 85.53% |
| 56 | 29531 | 27168 | 20470 | 6690 | 8 | 2363 | 75.35% |
| 58 | 29531 | 28040 | 20075 | 7960 | 5 | 1491 | 71.59% |
| 60 | 29531 | 26225 | 19717 | 6500 | 8 | 3306 | 75.18% |
| 62 | 29531 | 25685 | 19785 | 5889 | 11 | 3846 | 77.03% |
| 64 | 29531 | 21262 | 18865 | 2388 | 9 | 8269 | 88.73% |
| 65 | 29531 | 23970 | 20333 | 3629 | 8 | 5561 | 84.83% |
| 66 | 29531 | 21782 | 19667 | 2103 | 12 | 7749 | 90.29% |
| 67 | 29531 | 25980 | 19620 | 6356 | 4 | 3551 | 75.52% |
| 68 | 29531 | 26035 | 20032 | 5997 | 6 | 3496 | 76.94% |
| 69 | 29531 | 22979 | 20332 | 2627 | 20 | 6552 | 88.48% |
| 70 | 29531 | 26112 | 19943 | 6157 | 12 | 3419 | 76.37% |
| 71 | 29531 | 21050 | 18243 | 2790 | 17 | 8481 | 86.67% |
| 75 | 29531 | 20789 | 18556 | 2222 | 11 | 8742 | 89.26% |
| 77 | 29531 | 23673 | 19220 | 4433 | 20 | 5858 | 81.19% |
| 78 | 29531 | 20972 | 18739 | 2218 | 15 | 8559 | 89.35% |
| 80 | 29531 | 27437 | 20481 | 6949 | 7 | 2094 | 74.65% |
| 81 | 29531 | 26666 | 18170 | 8490 | 6 | 2865 | 68.14% |
| 82 | 29531 | 25670 | 19269 | 6392 | 9 | 3861 | 75.06% |
| 83 | 29531 | 24000 | 21092 | 2892 | 16 | 5531 | 87.88% |
| 84 | 29531 | 26475 | 21473 | 4989 | 13 | 3056 | 81.11% |
| 88 | 29531 | 25139 | 21344 | 3788 | 7 | 4392 | 84.90% |
| 92 | 29531 | 25406 | 19544 | 5850 | 12 | 4125 | 76.93% |
| 95 | 29531 | 24896 | 19426 | 5463 | 7 | 4635 | 78.03% |
| 96 | 29531 | 24899 | 21691 | 3195 | 13 | 4632 | 87.12% |
| 97 | 29531 | 26401 | 20056 | 6334 | 11 | 3130 | 75.97% |
| 98 | 29531 | 24686 | 23014 | 1663 | 9 | 4845 | 93.23% |
| 99 | 29531 | 22262 | 19613 | 2635 | 14 | 7269 | 88.10% |
| 100 | 29531 | 24081 | 20166 | 3902 | 13 | 5450 | 83.74% |
| 103 | 29531 | 23935 | 20931 | 2990 | 14 | 5596 | 87.45% |
| 104 | 29531 | 23871 | 21759 | 2096 | 16 | 5660 | 91.15% |
| 105 | 29531 | 24447 | 19538 | 4900 | 9 | 5084 | 79.92% |
| 106 | 29531 | 22019 | 20091 | 1912 | 16 | 7512 | 91.24% |
| 107 | 29531 | 22164 | 20381 | 1771 | 12 | 7367 | 91.96% |
| 108 | 29531 | 26053 | 19423 | 6619 | 11 | 3478 | 74.55% |
| 109 | 29531 | 25340 | 19593 | 5733 | 14 | 4191 | 77.32% |
| 110 | 29531 | 24066 | 19724 | 4329 | 13 | 5465 | 81.96% |
| 114 | 29531 | 24875 | 21881 | 2979 | 15 | 4656 | 87.96% |
| 115 | 29531 | 23844 | 22576 | 1249 | 19 | 5687 | 94.68% |
| 116 | 29531 | 25393 | 20891 | 4485 | 17 | 4138 | 82.27% |
| 117 | 29531 | 23202 | 20408 | 2777 | 17 | 6329 | 87.96% |
| 118 | 29531 | 25227 | 22558 | 2657 | 12 | 4304 | 89.42% |
| 119 | 29531 | 22599 | 18268 | 4318 | 13 | 6932 | 80.84% |
| 120 | 29531 | 22182 | 19873 | 2293 | 16 | 7349 | 89.59% |
| 121 | 29531 | 25779 | 18829 | 6940 | 10 | 3752 | 73.04% |
| 122 | 29531 | 23016 | 20764 | 2241 | 11 | 6515 | 90.22% |
| 123 | 29531 | 27382 | 17650 | 9729 | 3 | 2149 | 64.46% |
| 125 | 29531 | 24043 | 20228 | 3798 | 17 | 5488 | 84.13% |
| 126 | 29531 | 23322 | 20727 | 2578 | 17 | 6209 | 88.87% |
| 127 | 29531 | 23725 | 19443 | 4271 | 11 | 5806 | 81.95% |
| 132 | 29531 | 25776 | 22380 | 3379 | 17 | 3755 | 86.82% |
| 133 | 29531 | 22040 | 20003 | 2021 | 16 | 7491 | 90.76% |
| 134 | 29531 | 26459 | 19145 | 7299 | 15 | 3072 | 72.36% |
| 135 | 29531 | 22847 | 19046 | 3784 | 17 | 6684 | 83.36% |
| 136 | 29531 | 24427 | 19296 | 5118 | 13 | 5104 | 78.99% |
| 138 | 29531 | 24287 | 19037 | 5235 | 15 | 5244 | 78.38% |
| 139 | 29531 | 26414 | 17844 | 8564 | 6 | 3117 | 67.56% |
| 140 | 29531 | 24096 | 19604 | 4477 | 15 | 5435 | 81.36% |
| 141 | 29531 | 23346 | 21039 | 2288 | 19 | 6185 | 90.12% |
| 143 | 29531 | 20816 | 19139 | 1660 | 17 | 8715 | 91.94% |
| 145 | 29531 | 21801 | 19341 | 2438 | 22 | 7730 | 88.72% |
| 146 | 29531 | 21641 | 19866 | 1761 | 14 | 7890 | 91.80% |
| 147 | 29531 | 26479 | 16943 | 9528 | 8 | 3052 | 63.99% |
| 148 | 29531 | 26006 | 18094 | 7900 | 12 | 3525 | 69.58% |
| 149 | 29531 | 21980 | 19981 | 1982 | 17 | 7551 | 90.91% |
| 150 | 29531 | 23999 | 19599 | 4388 | 12 | 5532 | 81.67% |
| 151 | 29531 | 21356 | 19378 | 1962 | 16 | 8175 | 90.74% |
| 152 | 29531 | 21211 | 20083 | 1117 | 11 | 8320 | 94.68% |
| 153 | 29531 | 23918 | 20700 | 3201 | 17 | 5613 | 86.55% |
| 156 | 29531 | 24332 | 20538 | 3787 | 7 | 5199 | 84.41% |
| 157 | 29531 | 20687 | 19035 | 1634 | 18 | 8844 | 92.01% |
| 159 | 29531 | 25263 | 19481 | 5770 | 12 | 4268 | 77.11% |
| 160 | 29531 | 26668 | 20422 | 6236 | 10 | 2863 | 76.58% |
| 161 | 29531 | 24031 | 21222 | 2794 | 15 | 5500 | 88.31% |
| 162 | 29531 | 25122 | 18201 | 6914 | 7 | 4409 | 72.45% |
| 163 | 29531 | 21105 | 20264 | 832 | 9 | 8426 | 96.02% |
| 165 | 29531 | 25284 | 18521 | 6753 | 10 | 4247 | 73.25% |
| 166 | 29531 | 20753 | 18725 | 2015 | 13 | 8778 | 90.23% |
| 170 | 29531 | 23589 | 19799 | 3779 | 11 | 5942 | 83.93% |
| 172 | 29531 | 22928 | 20064 | 2855 | 9 | 6603 | 87.51% |
| 173 | 29531 | 24496 | 19498 | 4991 | 7 | 5035 | 79.60% |
| 174 | 29531 | 23549 | 18700 | 4837 | 12 | 5982 | 79.41% |
| 175 | 29531 | 22367 | 19802 | 2557 | 8 | 7164 | 88.53% |
| 176 | 29531 | 24340 | 18599 | 5729 | 12 | 5191 | 76.41% |
| 177 | 29531 | 21129 | 19070 | 2040 | 19 | 8402 | 90.26% |
| 179 | 29531 | 23998 | 19464 | 4524 | 10 | 5533 | 81.11% |
| 180 | 29531 | 21828 | 19438 | 2370 | 20 | 7703 | 89.05% |
| 181 | 29531 | 25392 | 20887 | 4495 | 10 | 4139 | 82.26% |
| 182 | 29531 | 22598 | 19237 | 3351 | 10 | 6933 | 85.13% |
| 183 | 29531 | 24770 | 18772 | 5989 | 9 | 4761 | 75.79% |
| 184 | 29531 | 21460 | 18429 | 3017 | 14 | 8071 | 85.88% |
| 186 | 29531 | 22111 | 19831 | 2271 | 9 | 7420 | 89.69% |
| 187 | 29531 | 24413 | 19985 | 4417 | 11 | 5118 | 81.86% |
| 188 | 29531 | 21207 | 19802 | 1391 | 14 | 8324 | 93.37% |
| 190 | 29531 | 24025 | 20309 | 3705 | 11 | 5506 | 84.53% |
| 191 | 29531 | 27530 | 20102 | 7416 | 12 | 2001 | 73.02% |
| 193 | 29531 | 26255 | 23263 | 2984 | 8 | 3276 | 88.60% |
| 196 | 29531 | 25343 | 20696 | 4642 | 5 | 4188 | 81.66% |
| 197 | 29531 | 25750 | 20176 | 5561 | 13 | 3781 | 78.35% |
| 198 | 29531 | 24092 | 20534 | 3538 | 20 | 5439 | 85.23% |
| 199 | 29531 | 25530 | 21877 | 3645 | 8 | 4001 | 85.69% |
| 200 | 29531 | 25452 | 21017 | 4425 | 10 | 4079 | 82.58% |
| 201 | 29531 | 21247 | 19202 | 2025 | 20 | 8284 | 90.38% |
| 205 | 29531 | 23372 | 20365 | 2996 | 11 | 6159 | 87.13% |
| 206 | 29531 | 21682 | 20102 | 1569 | 11 | 7849 | 92.71% |
| 215 | 29531 | 22512 | 21151 | 1347 | 14 | 7019 | 93.95% |
| 216 | 29531 | 20784 | 18971 | 1799 | 14 | 8747 | 91.28% |
