# Supplementary material for: Spike-Stalk Injection Method Causes Extensive Phenotypic and Genotypic Variations for Rice Germplasm
Source: Front Plant Sci. 2020 Sep 25;11:575373. doi: 10.3389/fpls.2020.575373 (PMC7546333; doi:10.3389/fpls.2020.575373)
Supplement: Supplementary file 8 [file Table_8.docx]

Supplementary Table 8 Statistics of genomic heterozygosity and mutation ratio of ERV1 inbred lines

| Sample | Depth | Coverage | Covered_bases | hete-SNPs | heterozygosity(10-4) | mu_num(n>20) | mu_ratio(10^-4^) |
| --- | --- | --- | --- | --- | --- | --- | --- |
| 1 | 3.74 | 6.1 | 22620327 | 4146 | 1.83 | 12683 | 5.61 |
| 2 | 2.71 | 5.3 | 19808563 | 1517 | 0.77 | 11738 | 5.93 |
| 3 | 3.88 | 6.7 | 24934905 | 6258 | 2.51 | 16002 | 6.42 |
| 4 | 2.25 | 3.7 | 13790456 | 1065 | 0.77 | 5387 | 3.91 |
| 5 | 2.88 | 5.7 | 21190342 | 2604 | 1.23 | 12557 | 5.93 |
| 6 | 3.98 | 6.8 | 25359640 | 5252 | 2.07 | 15904 | 6.27 |
| 7 | 4.54 | 7.2 | 26968784 | 5494 | 2.04 | 16684 | 6.19 |
| 8 | 4.14 | 7 | 25897692 | 4470 | 1.73 | 16176 | 6.25 |
| 9 | 3.66 | 6.5 | 24069504 | 3553 | 1.48 | 14787 | 6.14 |
| 10 | 5.15 | 7.5 | 27927953 | 7983 | 2.86 | 18293 | 6.55 |
| 12 | 2.77 | 5 | 18731935 | 1834 | 0.98 | 9851 | 5.26 |
| 13 | 2.1 | 4.2 | 15814069 | 985 | 0.62 | 9070 | 5.74 |
| 14 | 2.73 | 5.6 | 20787715 | 3132 | 1.51 | 12439 | 5.98 |
| 15 | 3.13 | 5.7 | 21205910 | 3350 | 1.58 | 13744 | 6.48 |
| 17 | 3.25 | 6.1 | 22780507 | 3271 | 1.44 | 13848 | 6.08 |
| 18 | 2.85 | 5.7 | 21338469 | 3165 | 1.48 | 12701 | 5.95 |
| 19 | 2.68 | 5.5 | 20608873 | 2966 | 1.44 | 11903 | 5.78 |
| 20 | 5.83 | 7.8 | 28894894 | 8660 | 3.00 | 18179 | 6.29 |
| 22 | 3.8 | 6.5 | 24117197 | 5098 | 2.11 | 15146 | 6.28 |
| 23 | 1.88 | 3.1 | 11459194 | 319 | 0.28 | 6734 | 5.88 |
| 24 | 4.94 | 7.2 | 26758317 | 6176 | 2.31 | 16788 | 6.27 |
| 25 | 4.52 | 6.9 | 25614804 | 5971 | 2.33 | 12747 | 4.98 |
| 27 | 1.96 | 3.4 | 12682628 | 453 | 0.36 | 6407 | 5.05 |
| 28 | 4.13 | 6.7 | 24970304 | 5623 | 2.25 | 16683 | 6.68 |
| 29 | 1.74 | 3.7 | 13679645 | 238 | 0.17 | 7485 | 5.47 |
| 30 | 4.16 | 6.5 | 24081719 | 7375 | 3.06 | 16886 | 7.01 |
| 31 | 3.03 | 6 | 22516437 | 3625 | 1.61 | 14710 | 6.53 |
| 33 | 2.05 | 4.6 | 17022693 | 960 | 0.56 | 10086 | 5.93 |
| 34 | 3.06 | 5.3 | 19552029 | 1590 | 0.81 | 10272 | 5.25 |
| 35 | 5.8 | 7.9 | 29581404 | 12064 | 4.08 | 19654 | 6.64 |
| 36 | 4.17 | 7.2 | 26691185 | 8174 | 3.06 | 16362 | 6.13 |
| 38 | 4.39 | 7 | 26226097 | 3707 | 1.41 | 16704 | 6.37 |
| 39 | 4.2 | 7.2 | 26736426 | 9346 | 3.50 | 17711 | 6.62 |
| 40 | 5.47 | 7.7 | 28525084 | 10356 | 3.63 | 19292 | 6.76 |
| 42 | 3.34 | 6.4 | 23966938 | 4115 | 1.72 | 14115 | 5.89 |
| 43 | 4.04 | 7.1 | 26267623 | 7255 | 2.76 | 16577 | 6.31 |
| 45 | 2.63 | 5.7 | 21069006 | 1439 | 0.68 | 12160 | 5.77 |
| 46 | 5.19 | 7.5 | 27746752 | 8695 | 3.13 | 18423 | 6.64 |
| 47 | 4.06 | 6.7 | 25071717 | 5443 | 2.17 | 15980 | 6.37 |
| 48 | 7.71 | 8.3 | 31026684 | 10336 | 3.33 | 20838 | 6.72 |
| 52 | 1.62 | 3.6 | 13404320 | 222 | 0.17 | 7803 | 5.82 |
| 55 | 2.88 | 6 | 22404347 | 2381 | 1.06 | 14363 | 6.41 |
| 56 | 2.94 | 6.5 | 24202447 | 2589 | 1.07 | 14282 | 5.90 |
| 58 | 3.44 | 7.2 | 26933636 | 5894 | 2.19 | 16880 | 6.27 |
| 59 | 4.24 | 7.7 | 28789886 | 6851 | 2.38 | 18246 | 6.34 |
| 60 | 3.01 | 6.9 | 25729311 | 4331 | 1.68 | 15346 | 5.96 |
| 61 | 2.35 | 5.6 | 20758802 | 1562 | 0.75 | 12043 | 5.80 |
| 62 | 1.87 | 4.5 | 16612578 | 568 | 0.34 | 10268 | 6.18 |
| 63 | 4 | 7.4 | 27640277 | 8764 | 3.17 | 18456 | 6.68 |
| 64 | 4.42 | 7.7 | 28791429 | 7128 | 2.48 | 18097 | 6.29 |
| 65 | 4.26 | 7.8 | 28889209 | 8819 | 3.05 | 18333 | 6.35 |
| 66 | 1.79 | 4.2 | 15789835 | 523 | 0.33 | 8863 | 5.61 |
| 67 | 2.58 | 6.3 | 23515796 | 2595 | 1.10 | 12851 | 5.46 |
| 68 | 3.13 | 6.4 | 23886329 | 4064 | 1.70 | 14268 | 5.97 |
| 69 | 2.03 | 5 | 18548871 | 906 | 0.49 | 10263 | 5.53 |
| 70 | 6.61 | 9 | 33529833 | 9413 | 2.81 | 20906 | 6.24 |
| 72 | 2.63 | 5.6 | 20822613 | 1910 | 0.92 | 10679 | 5.13 |
| 73 | 2.04 | 3.5 | 13168431 | 635 | 0.48 | 6728 | 5.11 |
| 74 | 2.46 | 5.1 | 18929364 | 1897 | 1.00 | 11303 | 5.97 |
| 76 | 2.82 | 5.4 | 20204985 | 2866 | 1.42 | 11646 | 5.76 |
| 77 | 2.2 | 4.7 | 17612320 | 1179 | 0.67 | 10043 | 5.70 |
| 78 | 4.16 | 6.5 | 24096853 | 5671 | 2.35 | 15670 | 6.50 |
| 79 | 2.62 | 5.3 | 19857105 | 3010 | 1.52 | 11933 | 6.01 |
| 80 | 2.55 | 5.1 | 18953480 | 1633 | 0.86 | 10893 | 5.75 |
| 81 | 2.3 | 3.3 | 12383587 | 1053 | 0.85 | 6644 | 5.37 |
| 82 | 3.24 | 6 | 22427292 | 3777 | 1.68 | 13626 | 6.08 |
| 83 | 2.99 | 5.8 | 21624213 | 4237 | 1.96 | 12821 | 5.93 |
| 84 | 4.41 | 7 | 25945824 | 6946 | 2.68 | 15828 | 6.10 |
| 86 | 2.72 | 4.5 | 16860702 | 1910 | 1.13 | 10043 | 5.96 |
| 87 | 2.48 | 4.6 | 17169359 | 1880 | 1.09 | 10305 | 6.00 |
| 88 | 3.99 | 6.4 | 23736926 | 4273 | 1.80 | 14347 | 6.04 |
| 89 | 4.25 | 6.7 | 24767982 | 4780 | 1.93 | 15711 | 6.34 |
| 90 | 3.89 | 6.5 | 24251678 | 6593 | 2.72 | 15331 | 6.32 |
| 91 | 3.05 | 5.8 | 21747195 | 3311 | 1.52 | 12664 | 5.82 |
| 92 | 5.12 | 7.2 | 26672094 | 5779 | 2.17 | 17126 | 6.42 |
| 93 | 5.7 | 7.5 | 28053783 | 8817 | 3.14 | 17407 | 6.20 |
| 94 | 4.78 | 6.2 | 23234109 | 5969 | 2.57 | 14677 | 6.32 |
| 95 | 8.79 | 8.7 | 32282250 | 13699 | 4.24 | 21502 | 6.66 |
| 96 | 5.46 | 8.8 | 32592255 | 9664 | 2.97 | 18739 | 5.75 |
| 97 | 3.3 | 7.3 | 27319379 | 4511 | 1.65 | 16671 | 6.10 |
| 98 | 1.93 | 4.9 | 18243605 | 657 | 0.36 | 10114 | 5.54 |
| 100 | 2.44 | 6 | 22480815 | 1833 | 0.82 | 12735 | 5.66 |
| 101 | 1.79 | 4.4 | 16567574 | 465 | 0.28 | 9344 | 5.64 |
| 102 | 2.25 | 5.6 | 20868213 | 1152 | 0.55 | 12265 | 5.88 |
| 103 | 2.44 | 6 | 22379553 | 2539 | 1.13 | 13272 | 5.93 |
| 104 | 2.2 | 5.6 | 20946165 | 1192 | 0.57 | 12115 | 5.78 |
| 105 | 2.38 | 5.8 | 21627831 | 1738 | 0.80 | 12721 | 5.88 |
| 106 | 2.58 | 6.3 | 23299396 | 2781 | 1.19 | 13832 | 5.94 |
| 107 | 1.9 | 3.5 | 13161407 | 529 | 0.40 | 6210 | 4.72 |
| 108 | 5.37 | 9.2 | 34374502 | 11419 | 3.32 | 20861 | 6.07 |
| 109 | 3.51 | 7.3 | 27094646 | 2754 | 1.02 | 15295 | 5.65 |
| 110 | 3.08 | 7.1 | 26310542 | 5984 | 2.27 | 16553 | 6.29 |
| 111 | 2.35 | 6 | 22354957 | 2103 | 0.94 | 13299 | 5.95 |
| 112 | 3.99 | 7.7 | 28830197 | 5334 | 1.85 | 17516 | 6.08 |
| 113 | 3.5 | 7.5 | 27953521 | 4265 | 1.53 | 16682 | 5.97 |
| 114 | 2.21 | 5.9 | 21896611 | 1519 | 0.69 | 11884 | 5.43 |
| 115 | 3.73 | 8 | 29772475 | 6803 | 2.28 | 17777 | 5.97 |
| 116 | 4.73 | 8.7 | 32258210 | 9734 | 3.02 | 19888 | 6.17 |
| 117 | 3.77 | 7.9 | 29568087 | 7382 | 2.50 | 17436 | 5.90 |
| 118 | 4.25 | 8.3 | 30795611 | 8890 | 2.89 | 19260 | 6.25 |
| 119 | 5.02 | 9 | 33513877 | 10492 | 3.13 | 20535 | 6.13 |
| 120 | 4.17 | 8 | 29934198 | 4751 | 1.59 | 18017 | 6.02 |
| 121 | 4.33 | 8.7 | 32514016 | 5748 | 1.77 | 18069 | 5.56 |
| 122 | 2.27 | 5.9 | 22078261 | 1651 | 0.75 | 11929 | 5.40 |
| 123 | 2.16 | 5.6 | 20896569 | 1039 | 0.50 | 11246 | 5.38 |
| 124 | 1.71 | 4.3 | 15865462 | 334 | 0.21 | 8465 | 5.34 |
| 125 | 2.28 | 6.1 | 22725844 | 1801 | 0.79 | 12216 | 5.38 |
| 126 | 2.07 | 5.4 | 19947193 | 1022 | 0.51 | 11289 | 5.66 |
| 127 | 2.68 | 6.8 | 25256290 | 3342 | 1.32 | 14262 | 5.65 |
| 128 | 2.48 | 6.3 | 23582833 | 1937 | 0.82 | 12598 | 5.34 |
| 129 | 2.53 | 6.6 | 24583453 | 2888 | 1.17 | 13390 | 5.45 |
| 130 | 2.98 | 7.2 | 26850193 | 3450 | 1.28 | 14362 | 5.35 |
| 131 | 4.64 | 9.2 | 34241653 | 10982 | 3.21 | 19380 | 5.66 |
| 132 | 3.3 | 7.9 | 29508068 | 5415 | 1.84 | 16068 | 5.45 |
| 133 | 1.84 | 4.9 | 18189103 | 732 | 0.40 | 9634 | 5.30 |
| 134 | 2.58 | 6.4 | 23759049 | 3341 | 1.41 | 13908 | 5.85 |
| 135 | 1.72 | 4.3 | 15944070 | 572 | 0.36 | 9064 | 5.68 |
| 136 | 2.15 | 5.7 | 21235094 | 1587 | 0.75 | 12102 | 5.70 |
| 137 | 1.95 | 5.2 | 19251730 | 786 | 0.41 | 10271 | 5.34 |
| 138 | 2.7 | 6.8 | 25491557 | 3549 | 1.39 | 13934 | 5.47 |
| 139 | 1.6 | 3.9 | 14603807 | 236 | 0.16 | 7829 | 5.36 |
| 140 | 3.23 | 7.8 | 28932006 | 6756 | 2.34 | 16340 | 5.65 |
| 141 | 1.96 | 5.2 | 19473892 | 603 | 0.31 | 10331 | 5.31 |
| 142 | 3.72 | 8 | 29894176 | 5699 | 1.91 | 16860 | 5.64 |
| 143 | 2.53 | 6.5 | 24153493 | 2880 | 1.19 | 13140 | 5.44 |
| 144 | 5.07 | 9.3 | 34541820 | 9059 | 2.62 | 19789 | 5.73 |
| 145 | 3.72 | 6.7 | 24879499 | 6409 | 2.58 | 16187 | 6.51 |
| 146 | 3.06 | 6.1 | 22831076 | 3733 | 1.64 | 13670 | 5.99 |
| 147 | 2.39 | 5.1 | 19069671 | 1510 | 0.79 | 11605 | 6.09 |
| 148 | 2.28 | 5 | 18520332 | 1542 | 0.83 | 10735 | 5.80 |
| 149 | 1.65 | 3.6 | 13307722 | 278 | 0.21 | 7448 | 5.60 |
| 150 | 1.89 | 4.2 | 15697107 | 522 | 0.33 | 9500 | 6.05 |
| 151 | 3.75 | 6.6 | 24684699 | 6311 | 2.56 | 16157 | 6.55 |
| 152 | 3.84 | 6.6 | 24578997 | 3374 | 1.37 | 15190 | 6.18 |
| 154 | 2.58 | 5.5 | 20660870 | 2497 | 1.21 | 12703 | 6.15 |
| 155 | 4.5 | 7.1 | 26322613 | 7633 | 2.90 | 17191 | 6.53 |
| 156 | 3.54 | 6.7 | 24772778 | 5853 | 2.36 | 15016 | 6.06 |
| 157 | 4.91 | 7.2 | 26751025 | 8204 | 3.07 | 18078 | 6.76 |
| 158 | 3.87 | 6.6 | 24661060 | 5421 | 2.20 | 16269 | 6.60 |
| 159 | 5.04 | 7 | 26235437 | 7019 | 2.68 | 18295 | 6.97 |
| 160 | 4.18 | 6.8 | 25218332 | 5317 | 2.11 | 16567 | 6.57 |
| 161 | 2.33 | 5.1 | 18893424 | 1304 | 0.69 | 11691 | 6.19 |
| 162 | 4.43 | 7.1 | 26266347 | 8459 | 3.22 | 16840 | 6.41 |
| 163 | 5.88 | 7.8 | 28892740 | 9573 | 3.31 | 18894 | 6.54 |
| 164 | 4.98 | 7.3 | 27353485 | 6663 | 2.44 | 18037 | 6.59 |
| 165 | 5.14 | 7.4 | 27402249 | 8991 | 3.28 | 18567 | 6.78 |
| 166 | 5.41 | 7.6 | 28118666 | 9908 | 3.52 | 18817 | 6.69 |
| 167 | 6.56 | 7.9 | 29595064 | 13535 | 4.57 | 20089 | 6.79 |
| 168 | 5.55 | 7.5 | 27796227 | 7934 | 2.85 | 18235 | 6.56 |
| 169 | 4.6 | 8.4 | 31327606 | 10606 | 3.39 | 19427 | 6.20 |
| 170 | 2.6 | 6 | 22475309 | 2130 | 0.95 | 13034 | 5.80 |
| 171 | 3.06 | 6.9 | 25572897 | 3399 | 1.33 | 14470 | 5.66 |
| 172 | 1.62 | 3.6 | 13347354 | 220 | 0.16 | 7420 | 5.56 |
| 173 | 2.09 | 5 | 18728222 | 954 | 0.51 | 10739 | 5.73 |
| 174 | 3.09 | 6.7 | 25062235 | 3697 | 1.48 | 14842 | 5.92 |
| 175 | 1.82 | 4.2 | 15657923 | 503 | 0.32 | 8906 | 5.69 |
| 176 | 3.3 | 7.1 | 26390511 | 5134 | 1.95 | 15670 | 5.94 |
| 177 | 3.55 | 7.4 | 27387385 | 4271 | 1.56 | 16474 | 6.02 |
| 178 | 3.25 | 7.1 | 26488307 | 5546 | 2.09 | 15931 | 6.01 |
| 179 | 3.64 | 7.5 | 27865003 | 5061 | 1.82 | 16353 | 5.87 |
| 180 | 3.77 | 7.7 | 28722994 | 6269 | 2.18 | 16412 | 5.71 |
| 181 | 2.46 | 5.9 | 22035437 | 2326 | 1.06 | 12276 | 5.57 |
| 182 | 2.21 | 5.3 | 19898783 | 1208 | 0.61 | 11495 | 5.78 |
| 183 | 2.72 | 6.3 | 23478804 | 2981 | 1.27 | 13623 | 5.80 |
| 184 | 3.35 | 7 | 26012492 | 4217 | 1.62 | 16114 | 6.19 |
| 185 | 2.59 | 6 | 22491067 | 2877 | 1.28 | 12967 | 5.77 |
| 187 | 2.01 | 4.9 | 18373821 | 863 | 0.47 | 9828 | 5.35 |
| 188 | 2.7 | 6.3 | 23386116 | 2768 | 1.18 | 13314 | 5.69 |
| 189 | 2.13 | 5.1 | 19140849 | 1301 | 0.68 | 10518 | 5.50 |
| 190 | 3.28 | 6.9 | 25515330 | 2324 | 0.91 | 14964 | 5.86 |
| 191 | 2.27 | 5.3 | 19885009 | 1260 | 0.63 | 11173 | 5.62 |
| 192 | 3.31 | 7.1 | 26270415 | 4744 | 1.81 | 15519 | 5.91 |
| 193 | 2.33 | 5.3 | 19578424 | 1002 | 0.51 | 10864 | 5.55 |
| 194 | 2.78 | 6.2 | 23228018 | 3452 | 1.49 | 13556 | 5.84 |
| 195 | 4.64 | 7.8 | 28990568 | 6300 | 2.17 | 17527 | 6.05 |
| 196 | 2.29 | 5.2 | 19324837 | 1204 | 0.62 | 10689 | 5.53 |
| 197 | 2.59 | 6 | 22238007 | 2782 | 1.25 | 12130 | 5.45 |
| 198 | 3.81 | 7.2 | 26970116 | 7084 | 2.63 | 16667 | 6.18 |
| 199 | 3.18 | 6.5 | 24259477 | 4093 | 1.69 | 14966 | 6.17 |
| 200 | 3.93 | 7.5 | 27906882 | 6543 | 2.34 | 16540 | 5.93 |
| 201 | 3.65 | 7.1 | 26579597 | 6273 | 2.36 | 15698 | 5.91 |
| 202 | 2.84 | 6.2 | 23072206 | 2460 | 1.07 | 13454 | 5.83 |
| 203 | 5.66 | 8.7 | 32554392 | 12404 | 3.81 | 19704 | 6.05 |
| 204 | 4.33 | 7.9 | 29430302 | 9297 | 3.16 | 17242 | 5.86 |
| 205 | 1.83 | 4.2 | 15652203 | 492 | 0.31 | 8611 | 5.50 |
| 206 | 2.89 | 6.5 | 24041632 | 3311 | 1.38 | 13921 | 5.79 |
| 207 | 3.09 | 6.6 | 24495849 | 4721 | 1.93 | 14675 | 5.99 |
| 208 | 3.84 | 7.3 | 27327174 | 8208 | 3.00 | 16828 | 6.16 |
| 209 | 4.5 | 7.8 | 29145511 | 7895 | 2.71 | 17772 | 6.10 |
| 210 | 3.17 | 6.6 | 24743871 | 5191 | 2.10 | 14422 | 5.83 |
| 211 | 1.73 | 3.9 | 14607627 | 273 | 0.19 | 7493 | 5.13 |
| 212 | 3.91 | 7.4 | 27468367 | 7390 | 2.69 | 17044 | 6.20 |
| 213 | 1.67 | 3.7 | 13751915 | 249 | 0.18 | 7533 | 5.48 |
| 214 | 3.96 | 7.4 | 27412434 | 6955 | 2.54 | 16919 | 6.17 |
| 215 | 3.56 | 7 | 25937844 | 5526 | 2.13 | 15452 | 5.96 |
| 216 | 4.68 | 7.8 | 29003299 | 9652 | 3.33 | 17530 | 6.04 |
