# Supplementary figures and images for: Spike-Stalk Injection Method Causes Extensive Phenotypic and Genotypic Variations for Rice Germplasm
Source: Front Plant Sci. 2020 Sep 25;11:575373. doi: 10.3389/fpls.2020.575373 (PMC7546333; doi:10.3389/fpls.2020.575373)

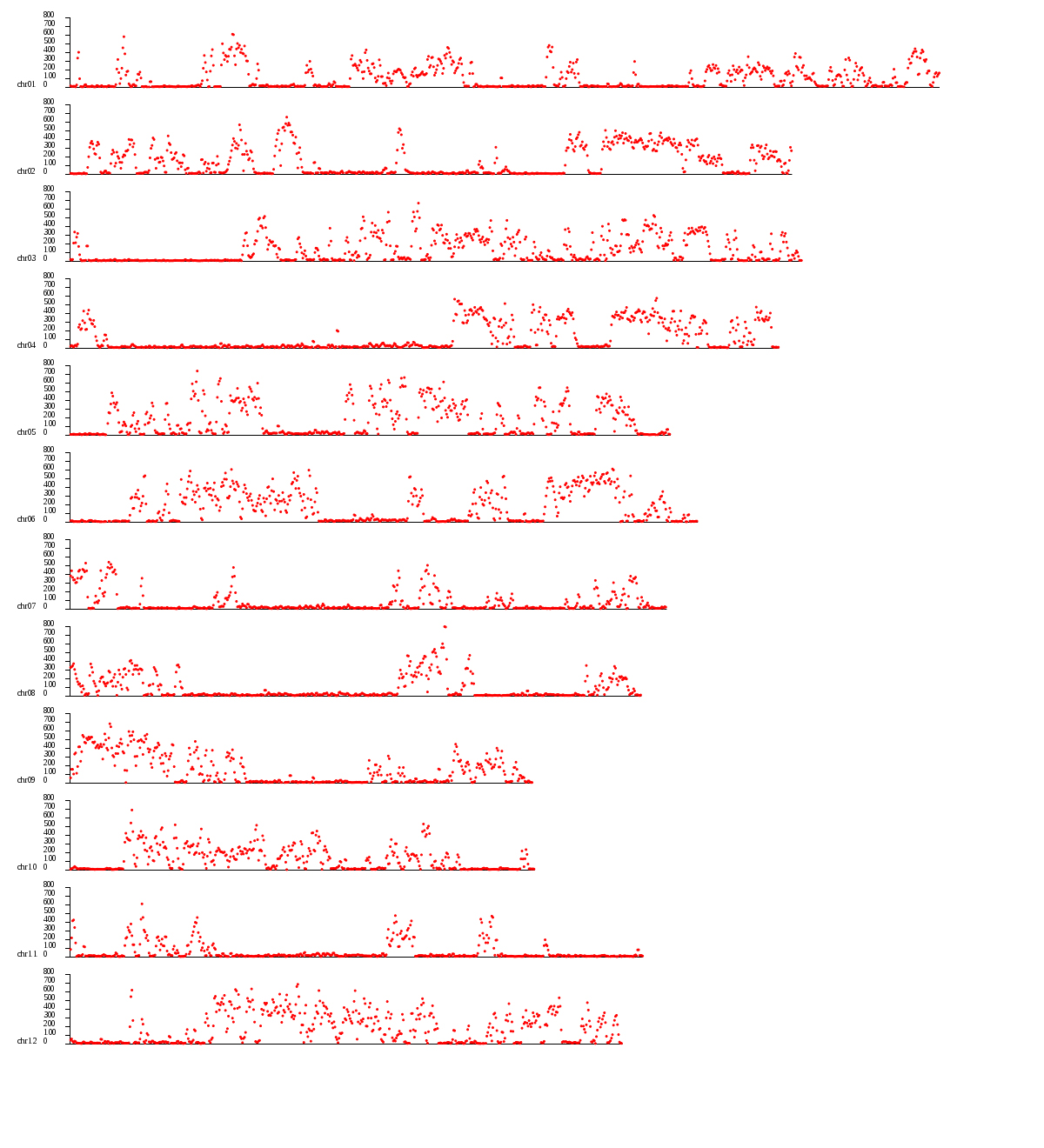

Supplement: Supplementary file 9 [file Image_1.tif]

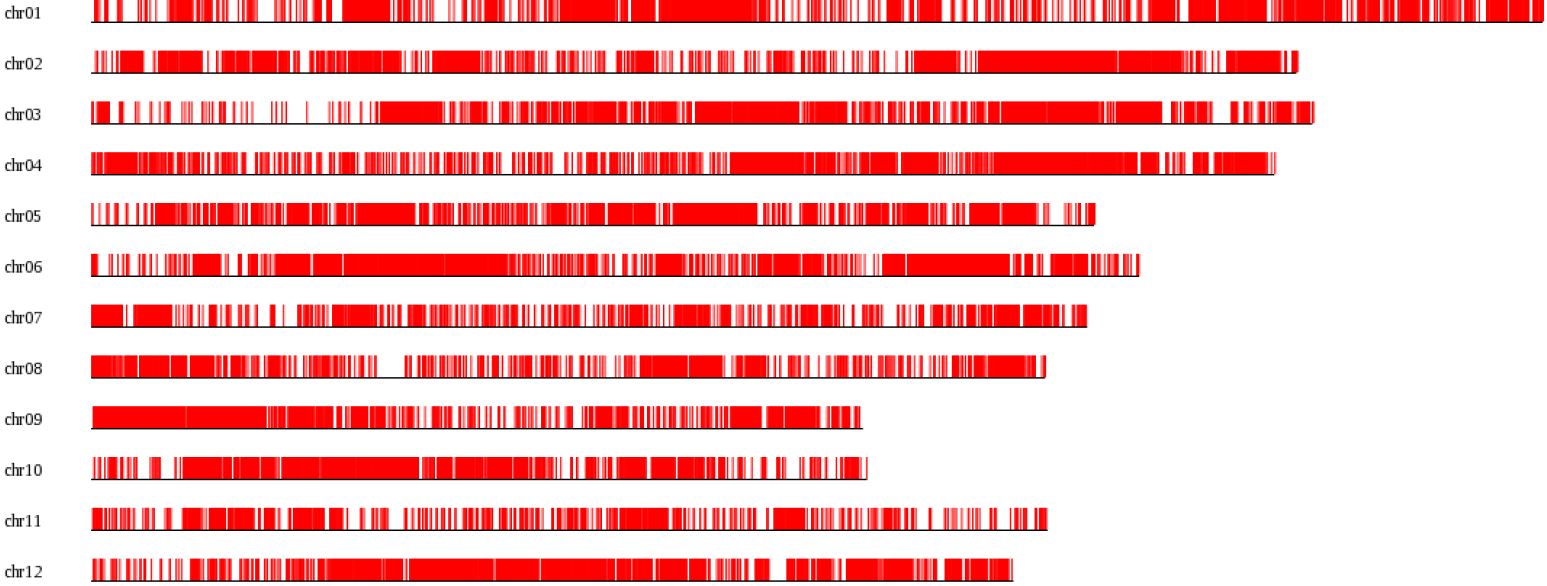

Supplement: Supplementary file 10 [file Image_2.tif]

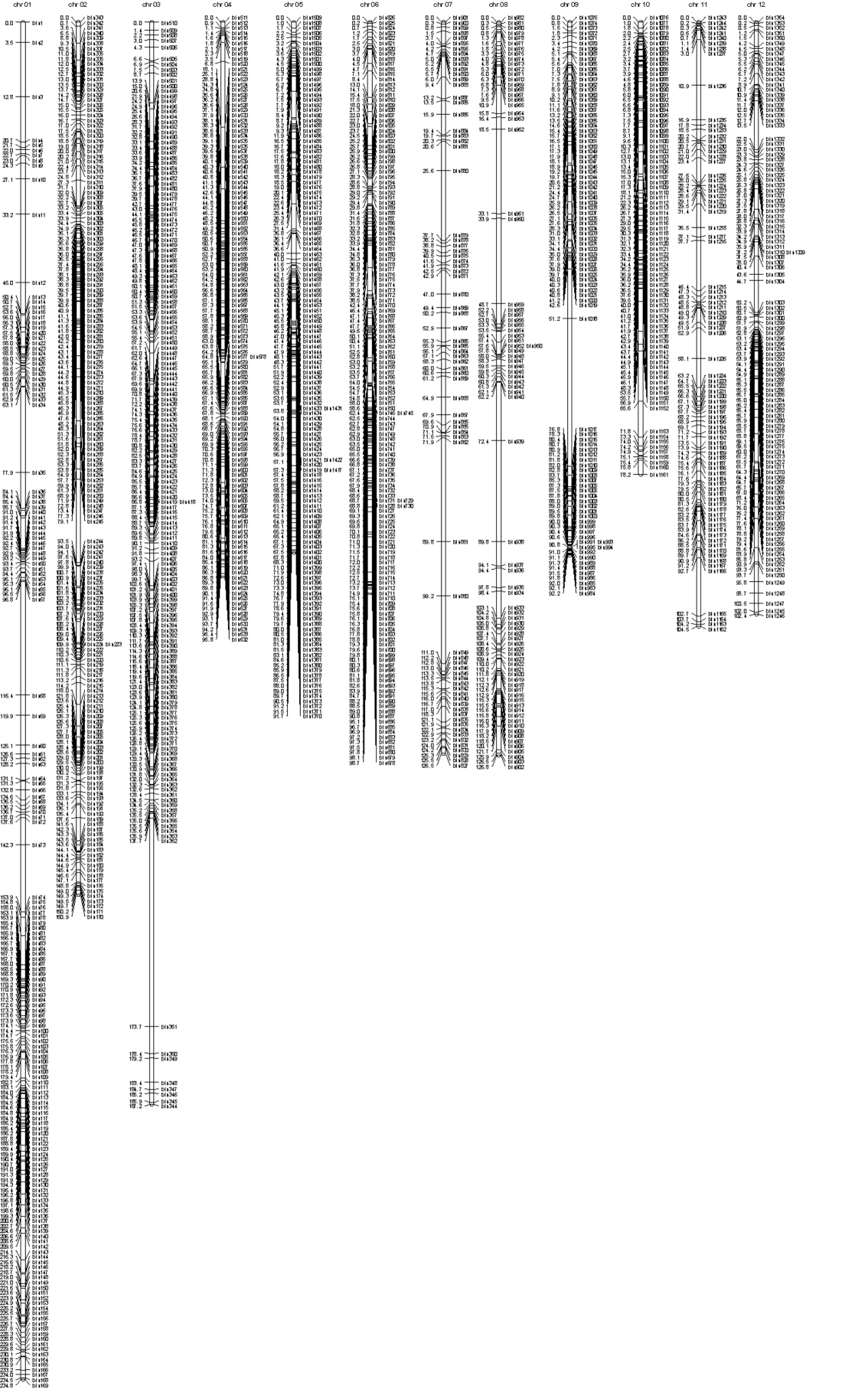

Supplement: Supplementary file 11 [file Image_3.tif]
